# Hyper- and hypoosmotic water permeability of bovine sperm as a function of age, breed, and ejaculate number: Implications for cryopreservation

**DOI:** 10.1371/journal.pone.0358553

**Published:** 2026-09-28

**Authors:** Mohsen Sharafi, Ali Mokhtassi-Bidgoli, Maajid Bhat, Patrick Vincent, Patrick Blondin, James D. Benson

**Affiliations:** 1 Semex Alliance, Guelph, Ontario, Canada; 2 Department of Biology, University of Saskatchewan, Saskatoon, Canada; 3 Molecular Diagnostic Laboratory, University of Montreal, Montreal, Canada; 4 Génome Québec, Montreal, Canada; University of Veterinary and Animal Sciences, PAKISTAN

## Abstract

The tolerance to cryoprotectant equilibration and the necessary cellular dehydration during equilibrium or quasi-equilibrium cooling that facilitates avoidance of intracellular ice is fundamentally connected to the ability of cells to transport water across the plasma membrane. Because of this, the relationship between the rate of water transport and post-thaw motility in bovine sperm has long been hypothesized, but has not been definitively established. Previous studies suggested that variability in bull sperm water permeability might explain post-thaw motility. Here we used a stopped-flow technique in which sperm were abruptly exposed to hypoosmotic and hyperosmotic media (220 mOsm/kg and 445 mOsm/kg final osmolalities, respectively) to critically evaluate this hypothesis and determine whether there is a relationship between the water permeability of fresh semen and the post-thaw motility rates in bull semen from various ejaculates, breeds, and age groups. We also evaluated additional possible predictive metrics of kinetic volume responses to anisosmotic medium, including the time to achieve 10%, 50% and 90% equilibrium volume (T10, T50, T90) in both hypoosmotic and hyperosmotic media. The total motility in the first of two ejaculates (Ejaculate 1) collected from each bull within a 10–15-minute interval was approximately 6.3% higher, and its progressive motility was about 8.1% higher than that of Ejaculate 2. Holstein bulls showed significantly higher total motility than Jersey bulls. No significant differences in either total or progressive motility of post-thaw bull semen were detected across the age groups of 12–24 months, 25–38 months, 40–57 months, and 69–116 months. Direct association analyses identified only weak relationships between water permeability and post-thaw motility, and none remained statistically significant after adjustment for multiple comparisons. The available sample size provided 80% power to detect absolute correlations of approximately 0.30 or greater. However, significant volume rectification was observed, with water permeability under hyperosmotic conditions being approximately 2% to 42% higher than under hypoosmotic conditions, depending on ejaculate number, breed, and age group. Principal component analysis indicated that *L*_p_ under hypoosmotic conditions, followed by *L*_p_ under hyperosmotic conditions, contributes most strongly to the separation of samples. Overall, the present paper demonstrates that both log-logistic and linear models provide reliable estimators of *L*_p_. Although *L*_p_ was not a robust predictor of post-thaw motility under the freezing conditions examined, these modeling approaches provide a quantitative framework for future studies investigating sperm osmotic behavior during cryopreservation.

## Introduction

Cryopreservation of bovine sperm is a critical component in all artificial insemination (AI) programs and has significantly contributed to the genetic improvement within dairy and beef agriculture [1,2]. Despite its widespread use, post-thaw sperm motility and viability are significantly reduced compared to fresh semen, thereby reducing the success of AI overall and costing industry hundreds of millions of dollars yearly [2–4]. To address this, decades of studies have focused on determining factors that influence post-thaw motility. For example, bull breed and age, and the collection interval, frequency, and season all have been shown to affect semen quality [1,5–11], and recently Hurri et al. [10] postulated that age, collection, and processing methods are important factors in determining the suitability of bull semen for commercial purposes. Despite advances in cryopreservation, post-thaw motility of bull sperm remains suboptimal. Bull-to-bull and ejaculate-to-ejaculate variability in post-thaw fertility means that AI centers must load straws with more sample than would be needed for fresh or optimally frozen semen [3,12,13]. The ability to better predict post-thaw quality would therefore improve AI product management and allow more efficient use of each ejaculate [3].

Post-thaw sperm quality is influenced by several interacting forms of cryoinjury, including oxidative stress, lipid peroxidation, mitochondrial dysfunction, DNA damage, and loss of plasma membrane integrity. For example, caffeic acid supplementation improved total and progressive motility, viability, plasma membrane integrity, and antioxidant status in frozen-thawed buffalo bull semen, demonstrating that preservation of membrane function and oxidative balance can substantially influence post-thaw sperm performance [14].

Because cells undergo many osmotic challenges during cryopreservation, the movement of water across cell membranes is critically important to the success of cryopreservation protocols for all cells and tissues. First, cells must undergo equilibration with and from high concentrations of permeating and nonpermeating cryoprotective solutes. Studies in several species have established the close connection between sperm viability, permeability to water and solutes, and cryoprotectant (CPA) equilibration protocols [15–20]. However, none of these studies directly connect membrane transport parameters and post-thaw motility on an individual ejaculate level, and their connections to post-thaw viability are mixed. Higher sperm motility may be associated with sustained water transport under small, near-isosmolar gradients, although the relationship between this response and measured water permeability values remains complex [16,17,21–24]. Noiles et al. [21] indicated that CPAs can significantly reduce the water permeability of sperm cells. Since motility relies on the intact function of subcellular components like mitochondria, which likely have lower water permeability than the plasma membrane, this reduction may have implications for preserving motility during cryopreservation [21]. Broader cryobiological evidence indicates that increasing CPA or solute concentration can reduce water and solute permeability in some cell systems [25]; however, whether these permeability changes directly improve post-thaw sperm motility remains unclear. The balance between altered membrane permeability, cryoprotective effects, and toxicity is crucial for optimizing cryopreservation outcomes [23,26–28]. Gilmore et al. [15,29] suggested that spermatozoa with relatively high water permeability values may be more capable of osmotic equilibration. However, permeability values measured above zero temperature are more relevant to membrane fluidity, osmotic tolerance, and equilibration with and from permeating CPA solutions than to direct prediction of membrane transport during subzero freezing. In addition, when comparing Table 3 and Figure 4 in the study by Agca et al. [30], a non-significant positive correlation was observed between the hydraulic conductivity parameter, *L*_p_, and the motility of Chimpanzee (*Pan troglodytes*) spermatozoa. It is important to note that these correlations were observed under varying osmotic conditions induced by different CPAs. More recently, García-Martínez et al. [31] emphasized that one of the conditions for minimizing injurious CPA exposure time is the rapid movement of water and CPA across the plasma membranes.

Towards a better understanding of how water and solute transport could be used to identify better freezing protocols in bovine sperm, Chaveiro et al. [16,17] established a stopped flow method for the identification of water and cryoprotectant permeability parameters in bovine sperm. This method relies on the use of the concentration dependent self-quenching property of the fluorophore carboxy fluorescein diacetate (CFDA) as a proxy for intracellular water volume. In their study, Chaveiro et al. [16,17] identified significant variability in water and CPA permeability and hypothesized that this variability might explain the wide variability in post-thaw viability [17]. Chaveiro et al. [17] did not directly assess this correlation, however, and they used a limited sample of seven Holstein donor bulls of unspecified age. Therefore, the relationship between fresh semen water permeability and post-thaw motility remains unresolved. Larger, multifactorial studies are therefore needed to determine whether variation in water permeability remains associated with post-thaw semen quality across different ejaculates, breeds, and age groups. This need is reinforced by evidence that cryopreservation outcomes also vary in response to membrane-protective and antioxidant interventions. For example, L-proline and fulvic acid supplementation produced concentration-dependent changes in post-thaw motility, viability, plasma membrane functionality, DNA integrity, and oxidative stress markers in buffalo bull semen [32].

Building upon this background, the aim of this study was to assess whether variations in water permeability of fresh semen correlate with post-thaw motility values in bull semen across different ejaculates, breeds, and age groups. We hypothesized that water permeability would be significantly positively correlated with post-thaw motility under the same freezing protocols. Thus, we sought to establish whether water permeability can serve as a reliable indicator of post-thaw motility, thereby aiding in the development of predictive models for cryopreservation success in bovine sperm. Additionally, because accurate fitting of the water transport differential equation requires extra data processing steps, we analyzed the relationship between more easily measured permeability dependent parameters and the fitted water permeability and assessed their ability to predict cryopreservation outcomes.

## Materials and methods

### Chemicals

Unless otherwise noted, Sigma (St. Louis, MO, USA) and Merck (Darmstadt, Germany) supplied all chemicals used in this study.

### Bulls and semen collection

The University of Saskatchewan Animal Care Committee (UAP002CatA2018) approved all animal experimental procedures. In this study, 83 semen samples from 60 bulls (48 Holstein, 10 Jersey, 1 Charolais, and 1 Angus) aged between 12 and 116 months were used. Ejaculates were collected using an artificial vagina from bulls regularly used for breeding purposes at Semex (Quebec, Canada). In commercial artificial insemination practice, two or three consecutive ejaculates are commonly collected at 10–15 minute intervals and pooled before routine semen processing. In the present study, the first and second ejaculates from 15 collection sessions were initially retained and processed separately to assess ejaculate-specific water permeability and post-thaw motility. Separate aliquots of Ejaculate 1 and Ejaculate 2 were cryopreserved and evaluated individually after thawing. Following the initial permeability measurements, additional aliquots from the two ejaculates were pooled and cryopreserved using the same extender, cooling, freezing, storage, and thawing procedures. Post-thaw motility was therefore evaluated separately for Ejaculate 1 and Ejaculate 2 and for the corresponding pooled sample. This design was used to distinguish biological variation associated with ejaculate order from variation introduced by pooling and cryopreservation. Processing the two ejaculates separately allowed direct comparison of their post-thaw characteristics, while evaluation of the pooled sample reproduced the routine commercial workflow. All separately frozen and pooled aliquots were subjected to the same cryopreservation protocol to minimize processing-related bias. This standardization was particularly important because extender composition and antioxidant status can influence membrane stability and post-thaw semen quality [33]. Nevertheless, pooling may average ejaculate-specific differences and was therefore analyzed as a distinct sample type rather than being substituted for the individual ejaculate measurements.

Semen samples were maintained in a water bath at 33 °C while a preliminary analysis of fresh semen was performed to measure concentration, motility, and morphology. Sperm concentration was determined using the Nucleocounter-SP100 cell counter (Chemometec, Denmark) according to the manufacturer’s instructions. An aliquot (50 µl) from each sample was mixed with 5 ml of reagent S100 in a sample cup. A cassette containing propidium iodide (PI) was filled with the mixture before being inserted into the Nucleocounter-SP100. The Nucleocounter-SP100 displayed the total cell count after 30 seconds. The total motility of fresh semen was assessed using the Sperm Class Analyzer (Microptic, Spain), which captures videos at 50 frames per second. Motility of the diluted sperm (2.5 µl) under a phase-contrast microscope (Nikon, Canada) was recorded using a pre-warmed chamber slide (37 °C, Life optic slide, 20 µm depth chamber). Morphology was determined using the Hancock method (Schäfer and Holzmann, 2000). In short, 50 µl of fresh sperm was put into a tube that already had 200 µl of Hancock solution in it. Hancock solution is made up of 150 ml of sodium saline solution, 150 ml of buffer solution, and 62.5 ml of formalin in 500 ml of double-distilled water. Then, 30 µl of the mixture was transferred onto a microscope slide and covered with a cover slip. Head, midpiece, and tail abnormalities were recorded under a phase-contrast microscope (Nikon, Canada) by counting a total of 200 sperm. Samples with standard quality metrics that included a concentration greater than 1 × 10^9^ sperm/ml, motility greater than 70%, and abnormal morphology less than 15% were selected for the experiments.

### Anisosmotic solutions, extender preparation, and cryopreservation

A tris-citric acid based medium was used to prepare the hyperosmotic and hypoosmotic solutions for measurement of cell volume responses. To prepare a hypoosmotic solution of 150 mOsmol/kg, the tris-citric acid based-medium was diluted with Milli-Q purified water. Hyperosmotic solutions were prepared by dissolving additional citric acid to achieve a solution of 600 mOsmol/kg. Osmolality was measured using a vapor pressure osmometer (Vapro 5520, Wescor, Logan, UT), with an accuracy of ±5 mOsmol/kg.

For the freezing experiments, a tris-based extender was prepared by dissolving 200 mmol/L Tris (hydroxymethyl-aminomethane), 66.7 mmol/L citric acid, and 55.5 mmol/L fructose. This extender was then mixed with 200 ml of fresh egg yolk and distilled water to produce 1 L of extender. Using the Tris-based extender, semen was diluted to a concentration of 20 × 10^6^ sperm/straw. The diluted sperm samples were cooled to 4 °C over a 4-hour equilibration period. Then 250 µL aliquots were distributed into 0.25 ml French straws and frozen in a Digitcool 007262 controlled-rate freezer (IMV, France) at −4 °C/min from 4 °C to −12 °C, −40 °C/min from −12 °C to −40 °C, and −50 °C/min from −40 °C to −140 °C. Finally, straws were plunged into liquid nitrogen. After at least 24 hours of storage, the frozen straws were individually thawed in a 37 °C water bath for 45 seconds prior to evaluation.

### Comparative analysis of post-thaw total and progressive motility

Motility was assessed using the Sperm Class Analyzer (Microptic, Spain). A 2.5 µl aliquot of diluted semen was placed on a pre-warmed chamber slide (37 °C, Life Optic slide, 20 µm depth chamber). Total and progressive motility were then determined using a phase-contrast microscope (Nikon, Canada) equipped with a 10 × objective at 33 °C.

### CFDA loading

A 5-carboxyfluorescein diacetate (CFDA) dye was added to the extended semen at a final concentration of 5 µM and incubated for 30 min at 37 °C. Excess CFDA was removed by centrifuging the cells for 6 min at 400 × g. The pellet was then resuspended in tris-citric acid-based medium [16,17].

### Stopped-flow measurements

Osmotic water permeability of bull sperm samples was measured by an SF-300X double mixing stopped-flow (KinTek Corporation, PA, USA) method as previously described [16,17]. Aliquots of sperm suspension (60 µl) were mixed with an equal volume of hypoosmotic or hyperosmotic solutions. Fluorescence was excited at 492 nm and detected using a 530 nm cut-off filter. The output of the photomultiplier tube was recorded every 1 x 10^−2^ s over 20 s and included both time and fluorescence measurements (Fig 1). Drawing from previous studies [17,29,34], a 20-second recording period was selected for the 150 mOsm treatment. Preliminary experiments demonstrated that sperm exposed to this hypoosmotic condition continued to swell for up to 120 s without reaching a stable fluorescence plateau. Consequently, extending the recording period would not have yielded a true equilibrium endpoint but would instead have increased the duration of exposure to severe hypoosmotic stress. Because prolonged exposure may introduce secondary biological responses, including membrane destabilization and regulatory volume changes that are unrelated to the initial passive water transport process, the 20 s recording window was selected to characterize the dynamic osmotic response under standardized experimental conditions. Therefore, the 150 mOsm treatment was used to characterize the kinetics of the osmotic swelling response rather than to estimate equilibrium cell volume, and all analyses were interpreted accordingly. Reproductive cells are highly susceptible to membrane-associated and oxidative injuries induced by prolonged environmental stress, emphasizing the importance of limiting experimental exposure to nonphysiological conditions during functional measurements [35]. The speed at which water enters or exits the cell directly influences the rate of cell water volume change. Changes in cell water volume alter the intracellular concentration of CFDA, which can be monitored through its concentration-dependent self-quenching behavior. Initially, fluorescence values were converted to cell volume (see Code 01 in S1 Code). Following Chaveiro et al. [17], the cell water volume, *w*, at each time point was calculated using the equation:

**Fig 1:**
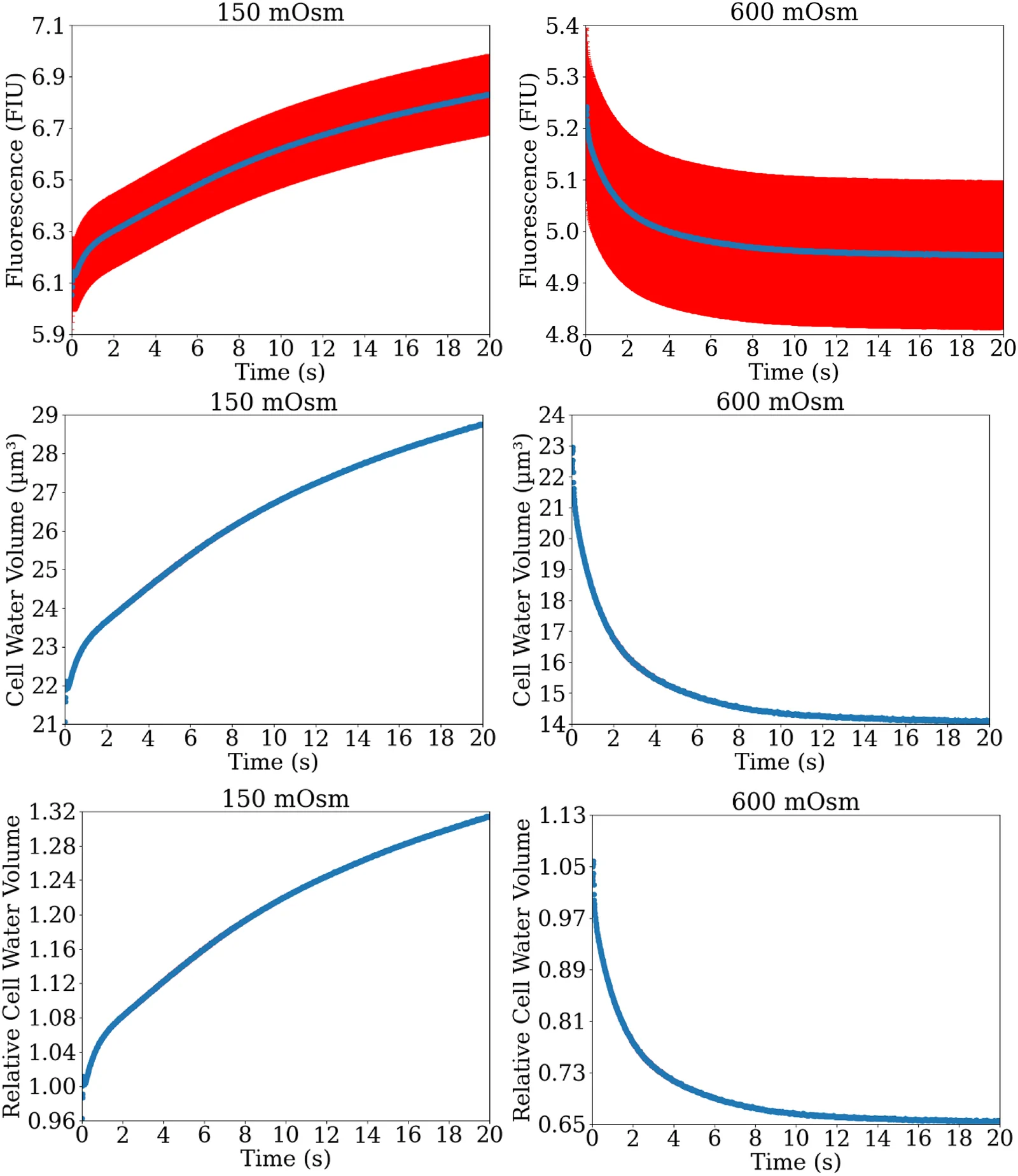
Fluorescence data and calculated water volume changes for two osmotic solutions (Mean of 83 samples in blue, standard errors in red). Sperm in isotonic Tris medium (290 mOsm) were mixed 1:1 with either 150 mOsm or 600 mOsm diluted or NaCl concentrated Tris medium and the resulting CFDA fluorescence intensity was measured every 10^−2^ s for 20 s.


$$\begin{array}{c} w(t)=\frac{I(t)-\left(\frac{I_{\text{0}}\pi_{\text{0}}-I_{\text{f}}\pi_{\text{f}}}{\pi_{\text{f}}-\pi_{\text{0}}}\right)}{\frac{\pi_{\text{f}}}{w(\text{0})}\left(\frac{I_{\text{0}}\pi_{\text{0}}-I_{\text{f}}\pi_{\text{f}}}{\pi_{\text{f}}-\pi_{\text{0}}}\right)}, \end{array}$$ Eq. 1

where *I(t)* is the fluorescence at time 𝑡; π_0_ is the initial (isotonic) osmolality (Tris-Hollande buffer), set at 290 mOsm; *π*_f_ is the final osmolality, which is 220 mOsm for hypoosmotic solutions (after 1:1 mixing of the isotonic medium with 150 mOsm mixing medium) and 445 mOsm for hyperosmotic solutions (after 1:1 mixing of the isotonic medium with 600 mOsm mixing medium); *w*_0_ is the initial cell volume, set to 21.9 µm^3^ [17]; *I*_*0*_ is the initial fluorescence, calculated by fitting a linear model to the first 100 time points (~1 s) and extracting the intercept. The endpoint fluorescence *I*_f_ was determined by fitting a linear model to the last 200 time points (~2 s) and using the slope and intercept to calculate the fluorescence at the final recorded time point. For the 150 mOsm hypoosmotic treatment, this value represents the selected recording endpoint rather than a confirmed equilibrium fluorescence, because the swelling response did not reach a stable plateau.

To estimate the hydraulic conductivity, *L*_p_, we discretized the water transport equation $w^{\prime }(t)=-L_{\text{p}}ART(\pi_{\text{f}}-\pi_{\text{i}}w_{\text{0}}w(t{)}^{-\text{1}})$ (c.f. Benson, [36]),


$$\begin{array}{c} w\left(t_{i}\right)-w\left(t_{i-\text{1}}\right)=-\Delta t\;L_{\text{p}}ART\left(\pi_{\text{f}}-\pi_{\text{i}}\frac{w_{\text{0}}}{w(t_{i-\text{1}})}\right), \end{array}$$ Eq. 2

which can be rearranged to find


$$\begin{array}{c} L_{\text{p}}=\frac{w\left(t_{i}\right)-w\left(t_{i-\text{1}}\right)}{\Delta t\;ART\left(\pi_{\text{f}}-\pi_{\text{i}}\frac{w_{\text{0}}}{w(t_{i-\text{1}})}\right)}, \end{array}$$ Eq. 3

where $t_{i}=i\Delta t$ is the time at the *i*th measurement with time interval Δ𝑡 = 1 x 10^−2^ s, 𝐴 is the surface area of the cell (145 µm^2^, [17]), 𝑅 is the gas constant (8.2085 x 10^−7^ µm^3^ atm K^-1^ mol^-1^), and *T* is the temperature (295.15 K). The $L_{\text{p}}$ was estimated using a nonlinear least-squares fitting procedure by minimizing the sum of squared errors (SSE) between the experimentally measured and modeled cell water volumes over time. The discretized form of the water transport equation was implemented, and optimization was performed using the Broyden–Fletcher–Goldfarb–Shanno (BFGS) algorithm. The best fit $L_{\text{p}}$value was determined, along with its standard error and 95% confidence interval, derived from the inverse Hessian matrix. The goodness of fit was assessed based on the resulting SSE, and a close agreement between the fitted and observed volumes indicated that the governing model appropriately described the experimental data under the given conditions (see Code 02 in S1 Code). To further assess the goodness of fit and model performance, both the coefficient of determination (R^2^) and Akaike Information Criterion (AIC) were calculated for each sample.

To evaluate the basic assumption regarding the self-quenching nature of CFDA and to determine whether the CFDA-derived water-volume responses were broadly consistent with near-ideal osmotic behavior, the estimated cell water volumes at three final osmolalities, 220 mOsm, 290 mOsm, and 445 mOsm, were plotted against the reciprocal of the extracellular osmolality, as described by the Boyle-van’t Hoff equation [31]:


$$\begin{array}{c} \frac{w}{w(\text{0})}=\left(\frac{\pi_{\text{0}}}{\pi_{\text{f}}}\right)\left(\text{1}-\frac{w_{\text{b}}}{w(\text{0})}\right)+\;\frac{w_{\text{b}}}{w(\text{0})} \end{array}$$ Eq. 4

where *w* is the cell water volume at final osmolality *π*_f_; *w*_0_ is the initial cell volume, set to 21.9 µm^3^; π_0_ is the initial (isotonic) osmolality (Tris-Hollande buffer), set at 290 mOsm; *π*_f_ is the final osmolality, which is 220 mOsm for hypoosmotic (150 mOsm) and 445 mOsm for hyperosmotic (600 mOsm) solutions; and *w*_b_ is the osmotically inactive fraction of the cell water volume. Because the standard Boyle–van’t Hoff relationship predicts zero relative water volume at a hypothetical infinite osmolality, the intercept was fixed at zero during regression fitting. In addition, because the 150 mOsm hypoosmotic treatment, corresponding to 220 mOsm final osmolality, did not reach a stable plateau, this analysis was not used to definitively establish complete osmotic equilibrium under that condition, but rather to assess whether the CFDA-derived water-volume responses followed the expected osmotic trend across the limited three-point osmolality range.

Next, to provide alternative metrics for evaluating sperm water permeability that do not require nonlinear regression, we determined the times required to reach 10%, 50%, and 90% of the projected equilibrium cell water volume, defined as *T10*, *T50*, *T90*, respectively. To do so, the fluorescence data were initially normalized using the following equation:


$$\begin{array}{c} f\left(t_{i}\right)=\;\text{1}-\;\frac{I\left(t_{i}\right)}{I_{\text{max}}\;}\;, \end{array}$$ Eq. 5

where *f* represents the normalized fluorescence data, falling within the range 0–1, *I* is the fluorescence at time *t*, and $I_{\text{max}}$ is the maximum fluorescence data point for the particular data set. In other words, the value of $I_{\text{max}}$ was determined individually for each data set corresponding to the hypoosmotic and hyperosmotic solutions, with measurements performed separately for each biological sample (bull).

A four-parameter log-logistic model was fitted to the normalized fluorescence data, f(t), as a function of time, *t* (see Code 03 in S1 Code). This model was selected because log-logistic and logistic-*t*ype nonlinear models are widely used for dose-response and time-response analyses, particularly when biological responses follow a sigmoidal saturation pattern [37,38]. The four-parameter log-logistic model provides a flexible sigmoidal form suitable for describing systems with lower and upper asymptotes, a slope parameter, and a midpoint or effective-response parameter [37,38]. The specific forms used here were adapted to estimate critical time points (T10, T50, T90), enabling direct comparison of the rate and extent of fluorescence response among treatments. This parameterization allows biologically meaningful interpretation of early-, median-, and late-phase fluorescence changes while providing a robust framework for nonlinear curve fitting [37,38]. Three nonlinear models, shown in Eqs. 6–8 were defined as follows:


$$\begin{array}{c} f(t)=\;f_{\text{min}}+\;\frac{f_{\text{max}}-\;f_{\text{min}}}{\text{1}+\text{exp}\left(b\left(\text{log}(t)-\text{log}(T\text{1}\text{0})+\frac{\text{1}}{b}\text{ log}(\text{9})\right)\right)}\;, \end{array}$$ Eq. 6

$$\begin{array}{c} f(t)=\;f_{\text{min}}+\;\frac{f_{\text{max}}-\;f_{\text{min}}}{\text{1}+\text{exp}\left(b\text{log}(t)-\text{log}(T\text{5}\text{0})\right)}\;, \end{array}$$ Eq. 7

$$\begin{array}{c} f(t)=\;f_{\text{min}}+\;\frac{f_{\text{max}}-f_{\text{min}}}{\text{1}+\text{exp}\left(b\;\left(\text{log}(t)-\text{log}(T\text{9}\text{0})-\frac{\text{1}}{b}\text{ log}(\text{9})\right)\right)}\;, \end{array}$$ Eq. 8

where *b* represents the slope of the curve, indicating the rate at which the normalized fluorescence intensity changes, and $f_{\text{min}}$ and $f_{\text{max}}$ denote the lower and upper limits of the curve, respectively, corresponding to the minimum and maximum normalized fluorescence intensities observed for each dataset.

### Statistical analysis

All data were analyzed using Python, employing various libraries for comprehensive analysis. Specifically, NumPy was used for numerical computations, pandas for data manipulation, SciPy for scientific computing, matplotlib and seaborn for data visualization, statsmodels for statistical modeling, and scikit-learn (sklearn) for regression applications (all code is provided in the supplementary files). For each factor (ejaculate, breed, and anisosmotic solution), which had two levels, comparisons were performed separately using Student’s t-test (assuming equal variances) or Welch’s t-test (when variances were unequal), to determine the corresponding p-values. K-means clustering was employed to categorize the bulls into four age classes, and p-values were determined using one-way analysis of variance (ANOVA). Residuals were evaluated to ensure they followed a normal distribution. Principal component analysis (PCA) was performed to identify patterns of variation among samples based on kinetic parameters and post-thaw motility traits. All variables were standardized prior to analysis. The first two principal components (PC1 and PC2) were retained for interpretation based on their contribution to total variance. PCA biplot was used to visualize the relationships among variables and the clustering of individual samples.

To complement the descriptive multivariate PCA and directly evaluate the primary study hypothesis, pairwise associations between hydraulic conductivity and post-thaw motility were assessed separately for total and progressive motility under hypoosmotic and hyperosmotic conditions. Pearson correlation coefficients with 95% confidence intervals and Spearman rank correlation coefficients were calculated. Because four primary *L*_p_ motility associations were tested, *P* values were additionally adjusted using the Benjamini-Hochberg false discovery rate procedure. Standardized ordinary least squares regression models were also fitted for each association, with both predictor and outcome variables standardized before analysis. Each of the 83 separately collected semen samples was treated as an observational unit because the samples were collected and experimentally processed separately and showed sample-specific variation in osmotic response and motility.

A sensitivity power analysis was performed for the primary two-sided Pearson correlation tests. With 83 observations and α = 0.05, the study had 80% power to detect an absolute correlation of approximately 0.303 or greater. The corresponding power was 44.2% for |r| = 0.20, 79.1% for |r| = 0.30, and 96.6% for |r| = 0.40. Therefore, the study was adequately powered to identify moderate to strong associations, whereas weak associations could not be excluded. Complete correlation, regression, power, and PCA loading results are provided in S4 to S8 Tables in S1 File.

Linear regression analysis was performed to evaluate the relationship between hydraulic conductivity (*L*_p_) and the reciprocal times (1/T50 and 1/T90) under anisosmotic conditions. For sample-level comparison of *L*_p_ between the 150 and 600 mOsm treatments, two-sided Z-tests were performed using the estimated *L*_p_ values and their corresponding standard errors for each condition.

## Results

Over all 83 data sets, fluorescence intensity (FI) increased from 6.09 ± 0.14 FIU to 6.87 ± 0.15 FIU when sperm were mixed 1:1 with 150 mOsm diluted Tris medium (mean ± SE), and FI decreased from 5.21 ± 0.15 FIU to 4.92 ± 0.14 FIU when sperm were mixed 1:1 with 600 mOsm Tris medium (Fig 1). Correspondingly, the calculated mean cell water volume was calculated to increase from 21.90 ± 0.05 µm^3^ to 28.86  ± 0.01 µm^3^ in the hypoosmotic (220 mOsm final osmolality) treatment and decrease from 21.90 ± 0.05 µm³ to 14.26  ± 0.01 µm³ in the hyperosmotic (445 mOsm final osmolality) treatment. This amounts to an osmotic range of 1.318 ± 0.001 and 0.642 ± 0.011 times their isosmotic water volume in 220 mOsm and 445 mOsm solutions, respectively (Fig 1). When the Boyle–van’t Hoff regression was fitted with the intercept fixed at zero, the relationship between relative water volume and relative inverse osmolality remained strongly linear (R^2^ = 0.99; Fig 2). The slope of the regression line (0.998) was close to unity, supporting proportional changes in CFDA-derived relative water volume across the limited three-point osmolality range tested. The hyperosmotic response reached an apparent plateau within the recording period; however, because the 150 mOsm hypoosmotic response did not reach a stable plateau, the hypoosmotic endpoint should be interpreted as an operational estimate rather than definitive evidence of complete osmotic equilibration under that condition. After analysis of the post-thaw parameters as a function of ejaculate number, age, and breed, we found that the post-thaw total and progressive motilities of Ejaculate 1 and Ejaculate 2 were significantly different (Fig 3). The total motility in Ejaculate 1 was approximately 6.3% higher, and its progressive motility was about 8.1% higher than in Ejaculate 2. Holstein bulls showed significantly higher total motility than Jersey bulls (p < 0.01; Fig 3B). While there is also a trend towards higher progressive motility in Holstein bulls, this difference is not statistically significant, with a p-value of 0.07. We found no significant differences in either total or progressive motility of post-thaw bull semen across the age groups of 12–24 months, 25–38 months, 40–57 months, and 69–116 months (Fig 3C). Although no statistically significant differences were observed among age groups, the overall mean total motility and progressive motility across all samples were 66.08% and 48.60%, respectively. Bulls between 3–5 years of age showed slightly higher post-thaw motility values compared to both younger and older bulls.

**Fig 2:**
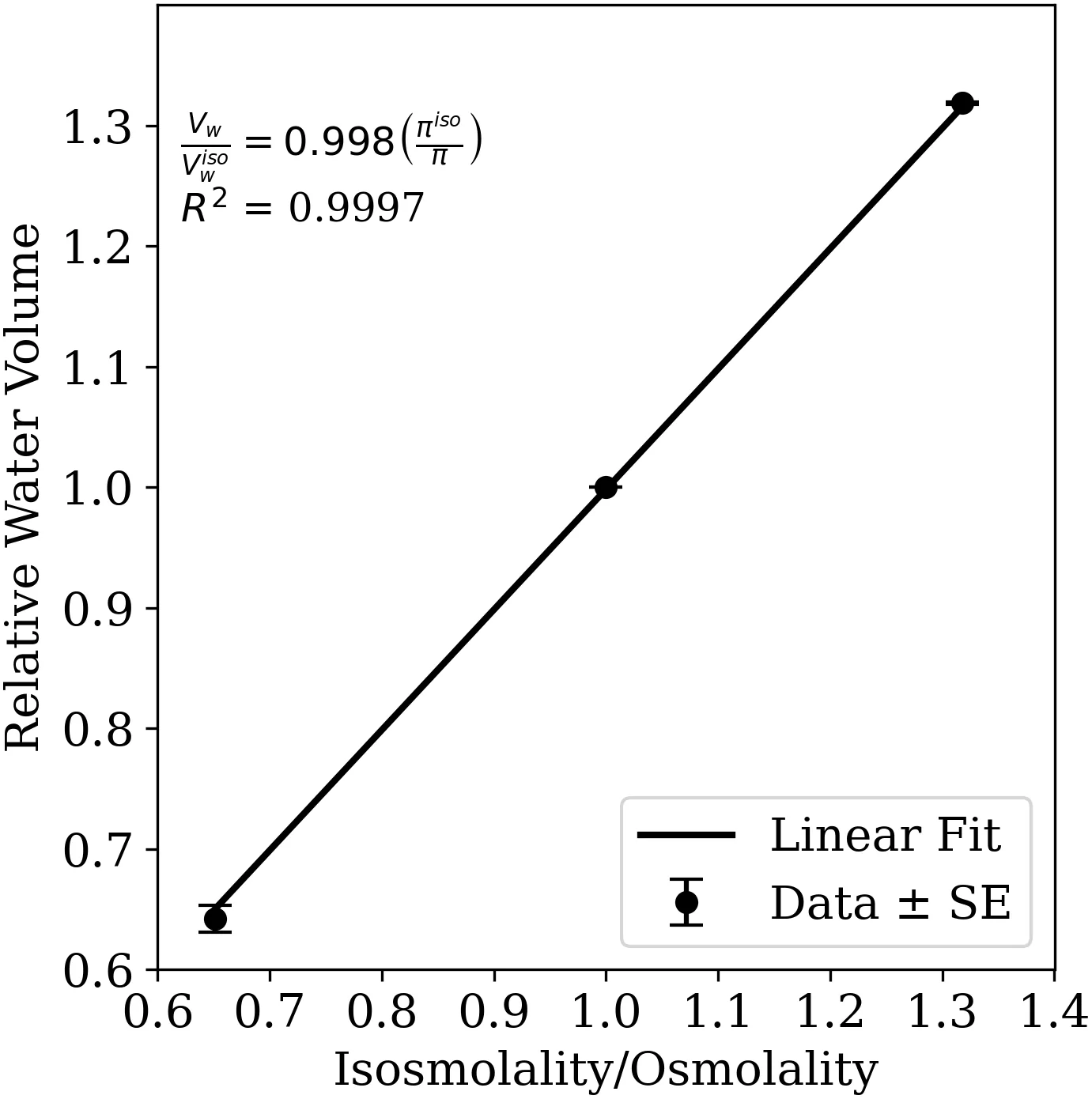
Boyle van’t Hoff plot showing the linear relationship between relative cell water volume and the osmotic ratio (isotonic osmolality / final osmolality) for bull sperm. Sperm were initially equilibrated in isotonic Tris medium (290 mOsm) and then exposed 1:1 to either 150 mOsm (DI water diluted; 220 mOsm final osmolality) or 600 mOsm (NaCl-concentrated; 445 mOsm final osmolality) Tris solutions. Data represent means ± SE from 83 individual samples.

**Fig 3:**
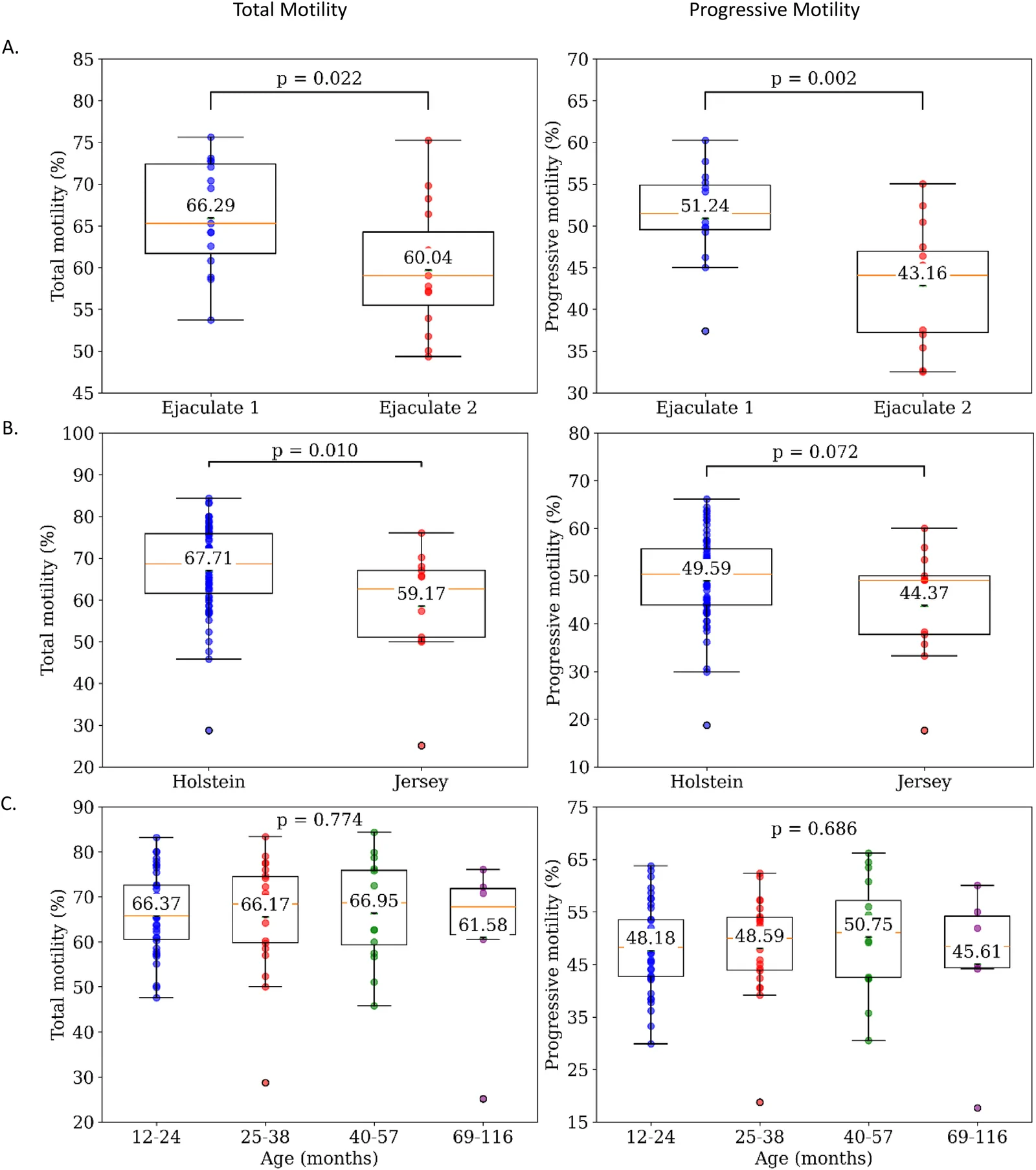
A. Comparative analysis of post-thaw total and progressive motility of bull semen in ejaculates 1 and 2 (n = 15). B. Comparative analysis of post-thaw total and progressive motility of bull semen between Holstein (n = 67) and Jersey (n = 13) bulls. C. Comparative analysis of post-thaw total and progressive motility of semen in bulls of different ages. In A and B, P values are according to Student’s t-test. In C, P values are from one-way ANOVA. Values in box plots are means. Boxes represent the interquartile range (IQR), spanning the first (Q1) to third (Q3) quartiles, with the median indicated by an orange line. Whiskers extend to 1.5 × the IQR from the quartiles. Outliers are shown as dots beyond the whiskers. All individual data points are shown.

When measuring osmotic response parameters like T10, T50, T90, and *L*_p_, Ejaculate 1 and Ejaculate 2 did not differ statistically in any osmotic response parameter after 1:1 mixing with hypoosmotic (150 mOsm) or hyperosmotic (600 mOsm) media (Fig 4). The p-values across the different comparisons range from 0.174 to 0.884 for T10 under hyperosmotic and hypoosmotic solutions, respectively.

**Fig 4:**
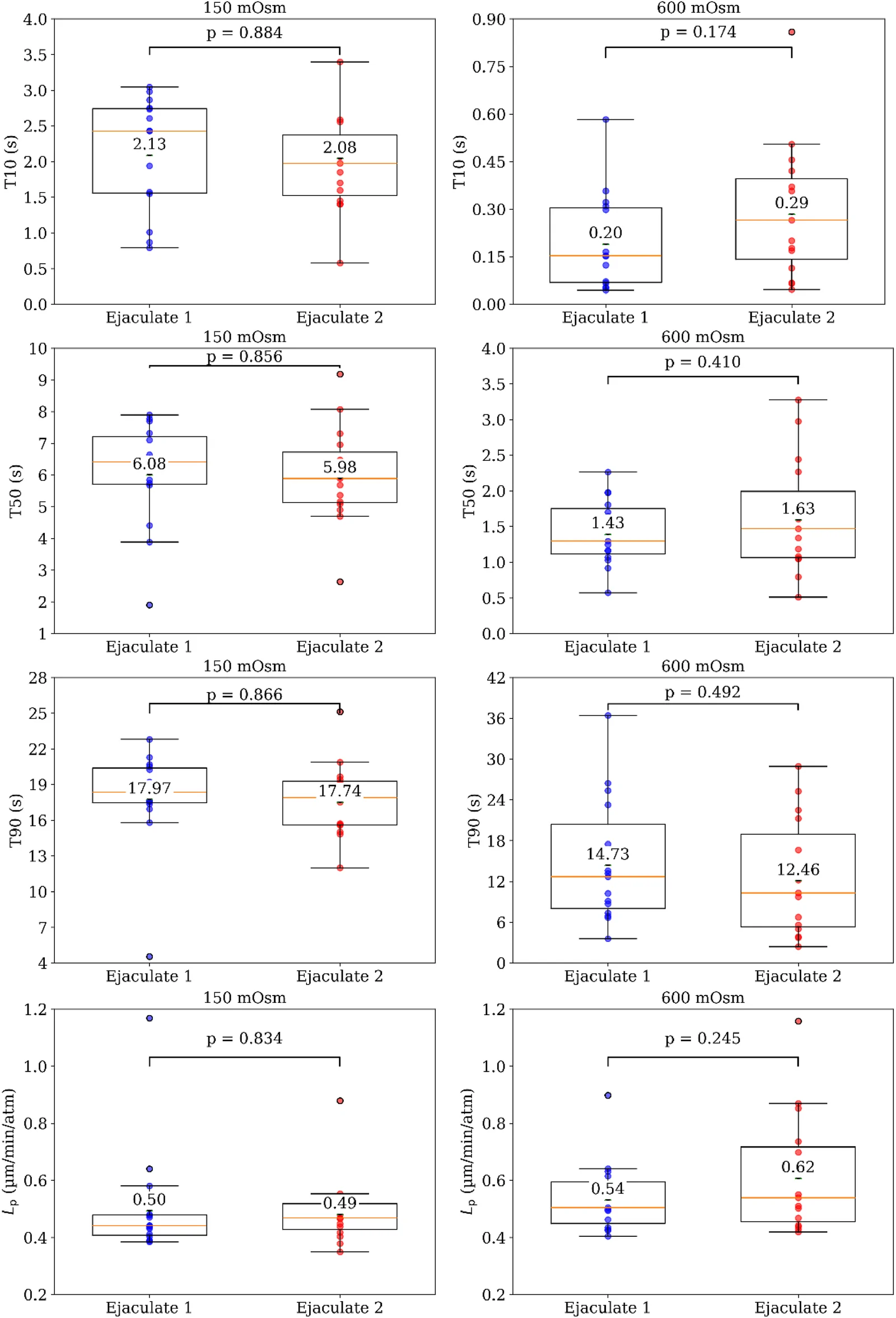
Comparative analysis of kinetic parameters (T10, T50, T90, L_p_) of bull semen in ejaculates 1 and 2 obtained after 1:1 mixing with hypoosmotic (150 mOsm; 220 mOsm final osmolality) and hyperosmotic (600 mOsm; 445 mOsm final osmolality) conditions. T10 = time to 10% cell water volume equilibrium, T50 = time to 50% cell water volume equilibrium, T90 = time to 90% cell water volume equilibrium, L_p_ = hydraulic conductivity. P values are according to Student’s t-test. Values in box plots are means. Boxes represent the interquartile range (IQR), spanning the first (Q1) to third (Q3) quartiles, with the median indicated by an orange line. Whiskers extend to 1.5 × the IQR from the quartiles. Outliers are shown as dots beyond the whiskers. The individual data points are shown.

Overall, there are no statistically significant differences between Holstein and Jersey breeds across all kinetic parameters (T10, T50, T90, and *L*_p_) after 1:1 mixing with hypoosmotic (150 mOsm) or hyperosmotic (600 mOsm) media, with most p-values exceeding 0.05 (Fig 5). However, there is a marginally non-significant difference in *L*_p_ under hypoosmotic conditions (p = 0.08), which suggests a trend toward different hydraulic conductivity between the breeds under these specific conditions. The mean *L*_p_ for Holstein bulls was 0.42 μm/min/atm, compared to 0.45 μm/min/atm for Jersey bulls.

**Fig 5:**
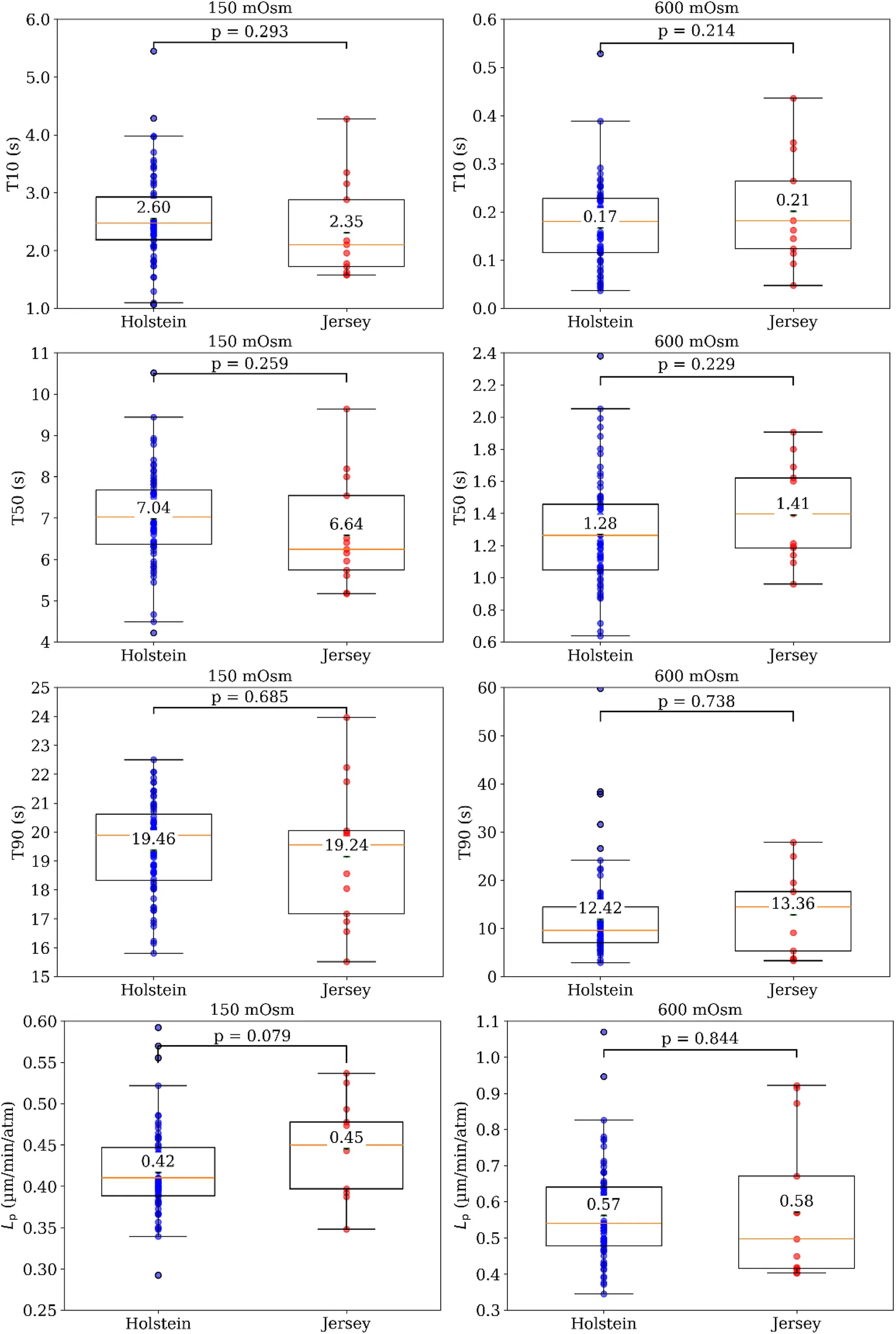
Comparative analysis of kinetic parameters (T10, T50, T90, L_p_) of bull semen from Holstein and Jersey breeds obtained after 1:1 mixing with hypoosmotic (150 mOsm) and hyperosmotic (600 mOsm) conditions. T10 = time to 10% cell water volume equilibrium, T50 = time to 50% cell water volume equilibrium, T90 = time to 90% cell water volume equilibrium, L_p_ = hydraulic conductivity. P values are according to Student’s t-test. Values in box plots are means. Boxes represent the interquartile range (IQR), spanning the first (Q1) to third (Q3) quartiles, with the median indicated by an orange line. Whiskers extend to 1.5 × the IQR from the quartiles. Outliers are shown as dots beyond the whiskers. The individual data points are shown.

We found no statistically significant differences in T10, T50, or *L*_p_ among the different age groups under both hypoosmotic and hyperosmotic conditions (Fig 6). However, we did find a significant difference in T90 under hypoosmotic conditions (p = 0.01), with bulls aged 25–38 months exhibiting a longer time to reach 90% cell volume compared to other age groups. A marginally non-significant difference for T90 under the hyperosmotic condition, *p* = 0.06, also supports this, potentially indicating age-related differences that require further study.

**Fig 6:**
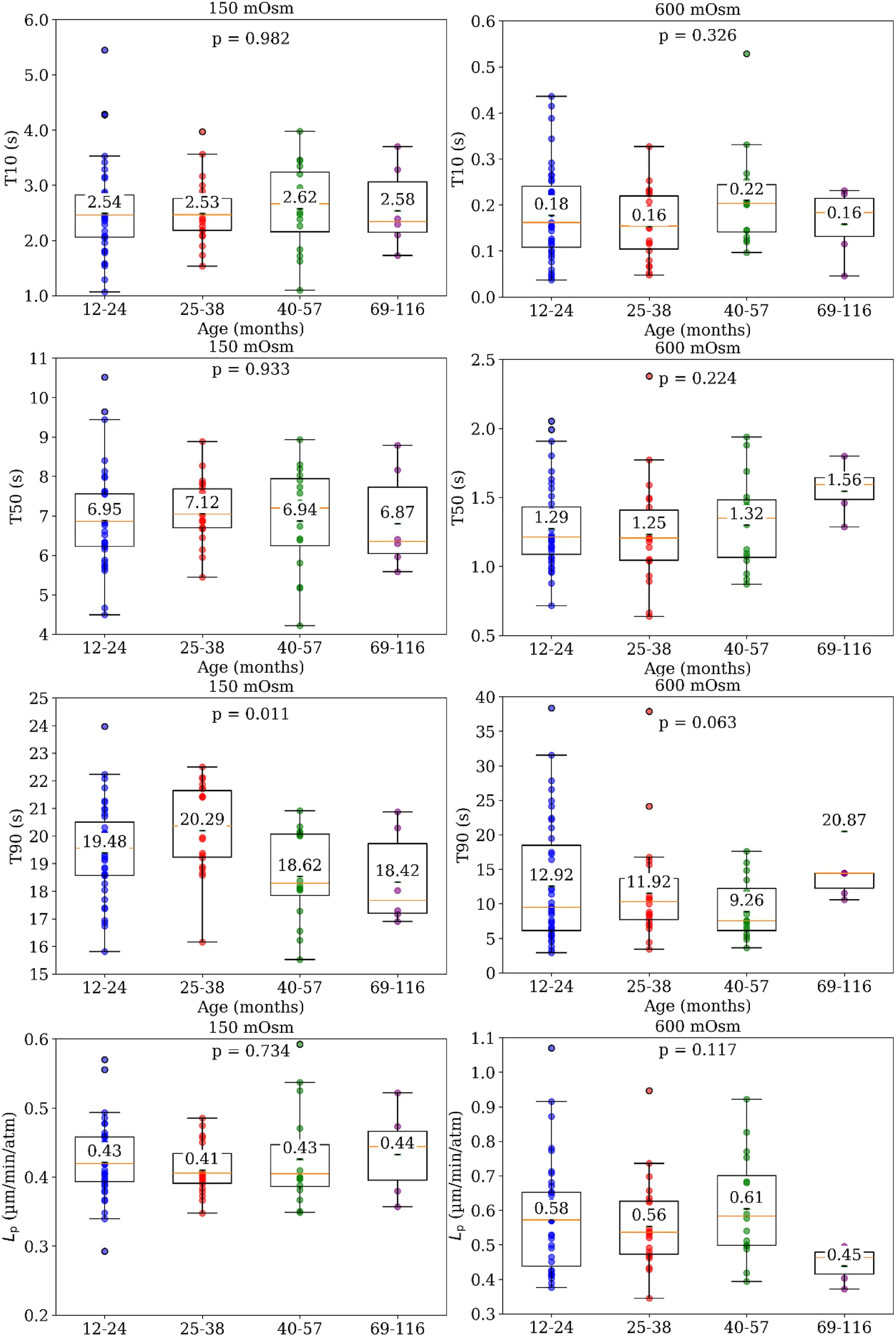
Comparative analysis of kinetic parameters (T10, T50, T90, L_p_) of semen in bulls of different ages under hypoosmotic (150 mOsm) and hyperosmotic (600 mOsm) conditions. T10 = time to 10% cell water volume equilibrium, T50 = time to 50% cell water volume equilibrium, T90 = time to 90% cell water volume equilibrium, Lp = hydraulic conductivity. P values are from one-way ANOVA. Values in box plots are means. Boxes represent the interquartile range (IQR), spanning the first (Q1) to third (Q3) quartiles, with the median indicated by an orange line. Whiskers extend to 1.5 × the IQR from the quartiles. Outliers are shown as dots beyond the whiskers. The individual data points are shown.

There were highly significant differences in the water permeability of bull sperm between hypoosmotic (220 mOsm final osmolality) and hyperosmotic (445 mOsm final osmolality) conditions, stratified by ejaculate number, breed, and age group (Fig 7). For Ejaculate 1, no significant difference was observed between osmotic treatments (*p* > 0.05), with only a 7.6% increase in *L*_p_ under hyperosmotic conditions. In contrast, Ejaculate 2 showed a significant 26.5% increase in *L*_p_ (*p* < 0.05). Among breeds, Holstein bulls exhibited a highly significant 35.7% increase in *L*_p_ (*p* < 0.01), while Jersey bulls showed a 28.9% increase (*p* < 0.05). Age-stratified analysis revealed significantly higher *L*_p_ values under hyperosmotic conditions in bulls aged 12–24 months (34.9% increase, *p* < 0.01), 25–38 months (36.6% increase, *p* < 0.01), and 40–57 months (41.9% increase, *p* < 0.01). However, bulls aged 69–116 months did not exhibit a significant difference, showing only a 2.3% increase in *L*_p_ (*p* > 0.05). These results indicate that hyperosmotic exposure generally increases sperm water permeability, with the effect modulated by ejaculate order, breed, and particularly age.

**Fig 7:**
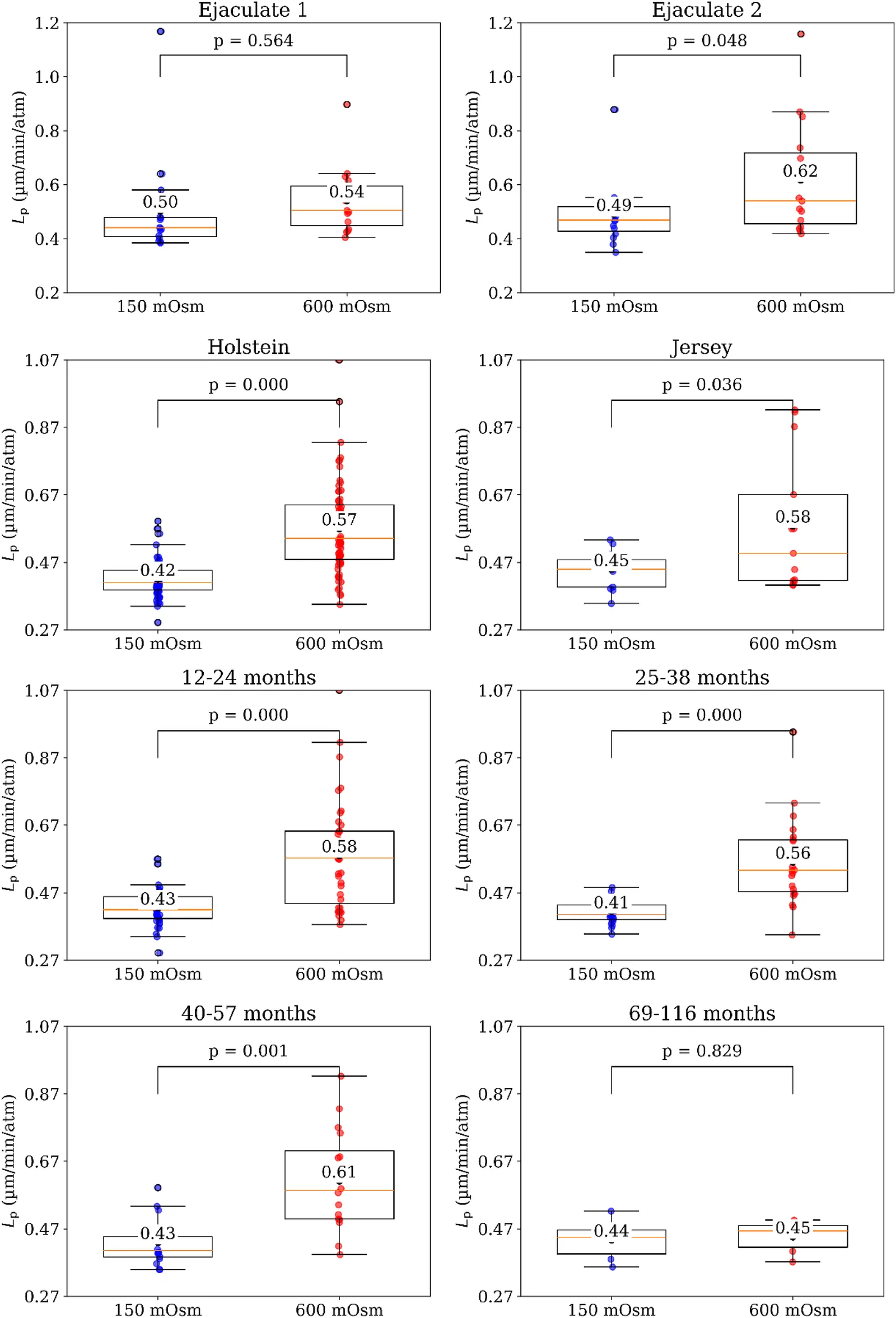
Comparative analysis of water permeability (L_p_) of bull sperm under 1:1 mixing with hypoosmotic (150 mOsm; 220 mOsm final osmolality) and hyperosmotic (600 mOsm; 445 mOsm final osmolality) conditions. L_p_ *measurements are stratified by ejaculate number, breed (Holstein vs. Jersey), and age group (in months). P values are according to Student’s t-tests. Values in box plots are means. Boxes represent the interquartile range (IQR), spanning the first (Q1) to third (Q3) quartiles, with the median indicated by an orange line. Whiskers extend to 1.5 × the IQR from the quartiles. Outliers are shown as dots beyond the whiskers. The individual data points are shown.*

Under hypoosmotic conditions (220 mOsm final osmolality), the water transport model exhibited excellent performance across 83 samples. The average sum of squared errors (SSE) was 185.06, with a standard error of 0.0025, and the 95% confidence interval for *L*_p_ ranged from 0.4194 to 0.4290 µm/min/atm. The mean coefficient of determination (R^2^) was 0.97, indicating very strong agreement between the observed and predicted values. The mean Akaike Information Criterion (AIC) was −5014.63, suggesting a highly efficient and well-fitting model (S1 Table in S1 File). Under hyperosmotic conditions (600 mOsm), model performance remained strong but showed slightly lower fit quality compared to the hypoosmotic condition. The average SSE was 279.85, with a standard error of 0.0071, and the 95% confidence interval for *L*_p_ ranged from 0.5603 to 0.5880 µm/min/atm. The mean R^2^ was 0.93, still indicating good agreement between the model and data. The mean AIC was −4324.39, reflecting a reliable yet relatively less optimal fit than that observed under hypoosmotic stress (S2 Table in S1 File).

The principal component analysis (PCA) biplot illustrates the multivariate relationships among measured variables and the clustering of individual semen samples based on osmotic response and kinetic traits. The first two principal components (PC1 and PC2) explain 34.83% and 22.26% of the total variance, respectively, accounting for 57.10% cumulatively (Fig 8; S7 Table in S1 File). Notably, *L*_p_ under hypoosmotic conditions shows the strongest loading along PC1, while *L*_p_ under hyperosmotic conditions dominates along PC2. This indicates that these two parameters contribute the most to the separation of samples in the PCA space, and they are major drivers of sample-level variation in osmotic water transport dynamics. However, post-thaw motility parameters—progressive motility and total motility— had small loadings on both axes, with PC1 loadings of −0.142 for both traits and PC2 loadings of 0.050 and 0.058, respectively (S8 Table in S1 File). Their positions near the origin therefore indicate that motility contributed little to the variation represented by the first two principal components, rather than providing a formal test of absence of association.

**Fig 8:**
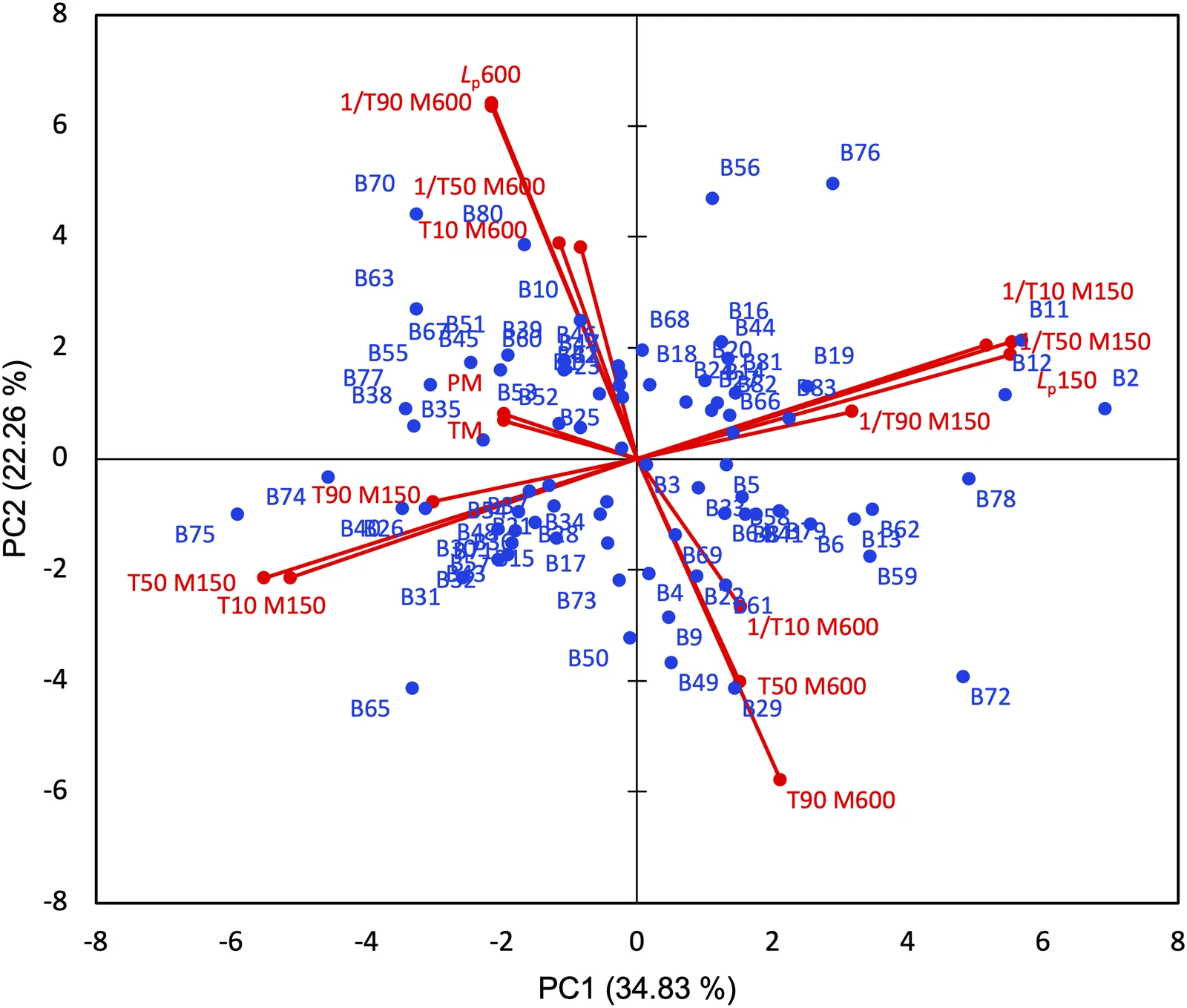
Principal component analysis (PCA) of kinetic parameters and motility traits in bull sperm under 1:1 mixing with hypoosmotic (M150 mOsm) and hyperosmotic (M600 mOsm) conditions. TM: Total Motility; PM: Progressive Motility; T10: Time to 10% cell water volume equilibrium; T50: Time to 50% cell water volume equilibrium; T90: Time to 90% cell water volume equilibrium; L_p_*: Hydraulic conductivity; 1/Ts: Reciprocals of times (1/T10, 1/T50, 1/T90). Arrows represent variable loadings, and blue dots represent semen samples.*

Direct association analyses were consistent with the limited contribution of motility in the PCA. No association remained statistically significant after false discovery rate adjustment (S4 Table in S1 File). Under hypoosmotic conditions, *L*_p_ showed weak negative associations with total motility, with Pearson *r* = −0.215, 95% CI −0.411 to 0.001, unadjusted *p* = 0.051, and with progressive motility, with Pearson *r* = −0.217, 95% CI −0.414 to −0.002, unadjusted *p* = 0.049. However, the corresponding false discovery rate adjusted *P* values were 0.103 for both associations. Under hyperosmotic conditions, associations of *L*_p_ with total motility and progressive motility were small and nonsignificant, with Pearson *r* values of 0.113 and 0.132, respectively. Standardized regression analyses produced the same pattern, with small effect estimates and no association meeting the false discovery rate adjusted significance threshold (S5 Table in S1 File).

The sensitivity analysis indicated that n = 83 provided 80% power to detect an absolute Pearson correlation of approximately 0.303. Power was 44.2% for a weak correlation of |*r*| = 0.20, 79.1% for |*r*| = 0.30, and 96.6% for |*r*| = 0.40 (S6 Table in S1 File). Thus, the study had adequate sensitivity for moderate to strong associations, but limited sensitivity for weak associations.

The linear regression equations for *L*_p_ versus the reciprocals of the times to reach 50% and 90% cell volumes (1/T50 and 1/T90) were determined for both hypoosmotic (220 mOsm final osmolality) and hyperosmotic (445 mOsm final osmolality) conditions (Fig 9). The regression models indicate that *L*_p_ values range from approximately 0.29 to 0.59 μm/min/atm under hypoosmotic conditions and from 0.35 to 1.07 μm/min/atm under hyperosmotic conditions. This suggests a faster rate of water expulsion under hyperosmotic stress than water influx under hypoosmotic stress in most bulls. However, this response was not universal, as two-sided Z-tests showed that approximately 17% of samples had higher *L*_p_ under hypoosmotic conditions and about 4% showed no significant difference between conditions (S3 Table in S1 File). Thus, higher hyperosmotic *L*_p_ should be interpreted as a predominant, but not uniform, pattern of osmotic response. These regression equations provide a reliable method for predicting *L*_p_ based on the reciprocal of the partial equilibration times (1/T50 and 1/T90). Given the strong correlation (R^2^ = 0.94 and R^2^ = 0.89) between *L*_p_ and the reciprocal times, these equations could be effectively utilized to estimate *L*_p_. Although *L*_p_ did not significantly correlate with post-thaw motility in this study, modeling hydraulic conductivity under osmotic stress conditions may still provide insights into mechanisms affecting sperm membrane function during cryopreservation. The absence of a robust association between hydraulic conductivity and post-thaw motility suggests that osmotic water transport alone does not explain the variability in sperm function after cryopreservation. Other processes, including oxidative stress, lipid peroxidation, mitochondrial dysfunction, and loss of plasma membrane integrity, may have a greater influence on motility after thawing. Evidence from turkey semen stored at 5 °C showed that glutathione and trehalose supplementation improved microscopic semen characteristics and oxidative stress parameters, illustrating that antioxidant balance and membrane protection can modify sperm quality independently of hydraulic conductivity [33]. Therefore, *L*_p_ should be interpreted as one component of a multifactorial response to semen preservation rather than as a stand-alone predictor of post-thaw motility. Modeling variation in hydraulic conductivity may provide mechanistic information about sperm osmotic behavior and membrane transport. However, because post-thaw motility is influenced by multiple oxidative, structural, and metabolic factors, *L*_p_ should not be considered an independent predictor of cryopreservation outcome.

**Fig 9:**
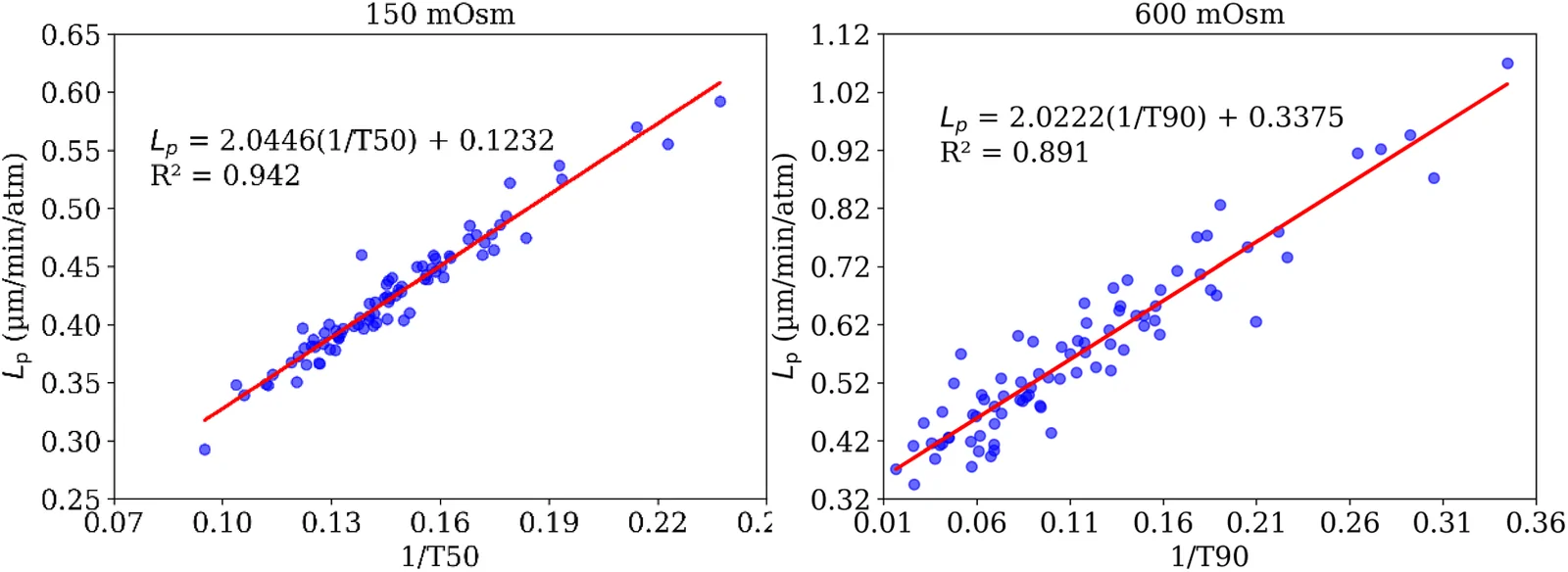
Linear regression analysis of hydraulic conductivity (L_p_) versus reciprocal times (1/T50 and 1/T90) under 1:1 mixing hypoosmotic (150 mOsm) and hyperosmotic (600 mOsm) conditions. T50: Time to 50% cell water volume equilibrium; T90: Time to 90% cell water volume equilibrium. The coefficient of determination (R^2^*) indicates the goodness of fit for the data under each condition.*

## Discussion

Overall, the findings from this study emphasize the importance of considering ejaculate source, breed, and age when assessing bull semen for post-thaw motility. Ejaculate 1, bulls within the 3–5 year age range, and Holstein bulls demonstrated significantly higher post-thaw motility. This suggests that these factors are very important in determining the quality and viability of semen after it has been frozen. Murphy et al. [1] found that bulls younger than one year exhibited the lowest semen production and sperm motility compared to bulls over one year of age. However, their study also indicated that collecting a second consecutive ejaculate does not result in a significant decrease in post-thaw sperm motility, suggesting this approach could be a useful way to increase the availability of sperm from young, genomically selected AI bulls that are in high demand. Hurri et al. [10] demonstrated that post-thaw semen quality in young bull ejaculates can vary significantly before they are deemed acceptable for commercial use, highlighting the importance of stringent quality control measures to ensure optimal fertility outcomes in artificial insemination programs. The review by Mandal et al. [39] highlights a clear relationship between bulls’ age and sperm motility, where an increase in age is associated with a decline in motility, potentially due to changes in fatty acid composition, particularly polyunsaturated fatty acids (PUFA) and docosahexaenoic acid (DHA). Hoflack et al. [6] argue that sperm motility differences between breeds, such as Holstein-Friesian (HF) and Belgian Blue (BB) bulls, reveal significantly higher total and progressive motility in HF bulls compared to BB bulls, as well as more efficient kinetic parameters in HF spermatozoa. These findings suggest a potential genetic basis for breed-specific variations in sperm motility, aligning with observations that even in unselected bull populations, motility differences may persist due to underlying genetic factors. Multiple genetic factors influence both total motility and progressive motility in Italian Holstein bulls, as revealed by Ramirez-Diaz et al. [40]. Based on the findings from Seyoum et al. [9], the post-thaw total motility and progressive motility percentages were significantly higher in HF bulls compared to Boran and Cross breeds.

The study's findings show that sperm kinetic parameters, including T10, T50, T90, and *L*_p_, were generally consistent across ejaculates, breeds, and age groups with most comparisons showing no statistically significant differences. However, some trends, such as breed-related differences in hydraulic conductivity and age-related variation in sperm motility, suggest areas for further investigation. Furthermore, bull sperm had highly significant differences in kinetic parameters (T10, T50, T90) between hypoosmotic (220 mOsm final osmolality) and hyperosmotic (445 mOsm final osmolality) conditions. The cells reached 10%, 50%, and 90% of their projected equilibrium volume more quickly under hyperosmotic conditions. This faster apparent equilibration is expected because a larger volume of water must move during hypoosmotic swelling than during hyperosmotic shrinkage. This direction-dependent difference in osmotic response is consistent with the concept of osmotic rectification, in which apparent hydraulic conductivity differs between water influx during hypoosmotic swelling and water efflux during hyperosmotic shrinkage. However, the direction of rectification observed in bull sperm here contrasts with Peckys et al. [41], who reported higher *L*_p_ during hypoosmotic water influx than during hyperosmotic water efflux in COS-7 cells. This discrepancy suggests that rectification is not a universal response, but may depend on cell type, membrane organization, cytoskeletal constraints, and the osmotic range tested. It is worth noting that the range of osmolalities tested here is much smaller than those tested for COS-7 cells. Our study examined sperm under final osmolality range of 220–445 mOsm (equivalent to a relative inverse osmolality range of 0.78 to 1.64), and Peckys et al looked at a range from ~157 mOsm to 602 mOsm (equivalent to a relative inverse osmolality range of 0.54 to 2.07), thus the Peckys study had a significant osmotic extension in both directions.

The direct association analyses clarify the interpretation of the negative primary finding. Weak inverse associations were observed between *L*_p_ under hypoosmotic conditions and both total and progressive motility, but these associations did not remain statistically significant after adjustment for multiple comparisons. Associations under hyperosmotic conditions were also small. These results were consistent with the PCA, in which motility variables had small loadings on the first two principal components and were positioned close to the origin. Importantly, PCA is descriptive and does not itself constitute a formal test of association. The correlation and standardized regression analyses therefore provide the direct statistical assessment of the relationship between *L*_p_ and post-thaw motility.

The sensitivity power analysis further defines the limits of this conclusion. The sample size provided 80% power to detect absolute correlations of approximately 0.303 and more than 96% power to detect correlations of 0.40. Therefore, the data do not support a moderate or strong relationship between fresh sperm hydraulic conductivity and post-thaw motility under the freezing protocol used here. However, power was only 44.2% for an association of |*r*| = 0.20, and weak relationships cannot be excluded. Future studies with larger sample sizes and repeated collections explicitly linked to individual bull identity would be required to resolve such small effects.

Overall, these findings highlight the stability of fresh sperm kinetic properties under different conditions, but they also point out potential underlying factors, such as breed, age, and osmotic pressure, that may subtly influence semen quality and warrant further study. *L*_p_ values range from approximately 0.29 to 1.07 μm/min/atm. The slightly higher *L*_p_ in Jersey bulls compared to Holstein bulls may reflect subtle differences in sperm membrane characteristics, morphology, osmotically inactive volume, or osmotic regulation mechanisms. Various methods, such as electronic particle counters, stopped-flow fluorometry, and differential scanning calorimetry, can measure *L*_p_. For this purpose, we used stopped-flow fluorometry in the present study. Chaveiro et al. [16,17] also used stopped-flow fluorometry to find out the *L*_p_ of bull spermatozoa with and without different CPAs. In one study [17], at 22 °C, the *L*_p_ in the absence of CPAs ranged between 0.22 and 0.33 μm/min/atm, whereas in another study [16], the *L*_p_ was reported to be 0.69 μm/min/atm under similar conditions. The authors suggest that these variations in *L*_p_ might reflect breed differences, with Holstein-Friesian bulls exhibiting a lower *L*_p_ compared to crossbreed bulls.

A complex interplay of factors, including membrane composition and structure, osmotically inactive volume fraction, the presence and activity of aquaporins, osmotic gradients, temperature, the use of cryoprotective agents, genetic variations, and cellular regulatory mechanisms, influences the dynamics of cell membrane *L*_p_. Understanding these dynamics is crucial in fields such as reproductive biology, cryopreservation, and cellular physiology, where maintaining proper water balance and protecting cell integrity are essential. Efficient sperm volume regulation is essential for normal sperm function. Prior to ejaculation, mammalian sperm are typically quiescent in the relatively hyperosmotic environment of the male reproductive tract, exhibiting minimal motility. However, upon entering the relatively hypoosmotic environment of the female reproductive tract during copulation, sperm experience a rapid activation of motility. This activation is closely linked to osmotic changes that trigger regulatory volume decrease (RVD) mechanisms, which protect sperm from harmful cell swelling and ensure their survival and functionality. As noted in recent studies, proteins like aquaporins, particularly AQP3, play a crucial role in facilitating these volume-regulatory processes by enabling rapid water movement across the sperm membrane, thereby optimizing sperm behavior under physiological conditions [42].

We note that this study was not carried out to research the direct effects of varying osmotic conditions on post-thaw motility rates since the osmotic conditions used when determining post-thaw motility were similar for all the samples. While the current work has focused on the water permeability of fresh semen under both hypoosmotic and hyperosmotic conditions, most studies are about osmotic tolerance and the direct effects of varying osmotic conditions on motility. For instance, the results from Gilmore et al. [29] showed that boar sperm cells can only tolerate volume deviations of 0.97 and 1.02 times their isosmotic volume while maintaining over 70% motility. Guthrie et al. [34] estimated that bull spermatozoa retain 90% of their isosmotic motility when their total cell volume is maintained within 92–103% of the isosmotic level, and approximately 50% of their isosmotic motility in the ranges evaluated in this study. Moreover, Liu and Foote [43] showed that >70% of sperm were intact after >30 minute exposures to 500 mOsm, and ~50% of sperm were intact after >30 minute exposures to 200 mOsm solutions. Based on these osmotic tolerance data, sperm were expected to remain largely membrane-intact within the relatively brief and limited final osmolality range tested here, although direct membrane-integrity measurements under these exact conditions were not performed. In our experiment, cell water volume was quantitatively assessed, and the zero-intercept Boyle-van’t Hoff regression suggested predictable and linear adjustments in CFDA-derived water volume across the three tested osmolalities. However, because the 150 mOsm hypoosmotic response did not reach a stable plateau even during extended recordings, this analysis should be interpreted as evidence of osmotic consistency over the tested range rather than definitive proof of complete equilibration. These findings are consistent with those of Chaveiro et al. [17], who reported an osmotic response range of approximately 1.30 and 0.65 times the isosmotic water volume when bull sperm cells were exposed to 230 mOsm and 455 mOsm solutions, respectively.

It is important to note that the present analysis was based on changes in cell water volume estimated from CFDA fluorescence, rather than total cell volume. Therefore, the Boyle–van’t Hoff analysis should not be used to estimate the total osmotically inactive volume fraction or directly compared with values reported in studies based on total sperm cell volume, such as Guthrie et al. [34]. Instead, the present results indicate that the osmotically responsive water fraction changed predictably across the tested osmotic range.

## Conclusion

This study examined the relationship between water permeability and post-thaw motility in bull semen, considering various kinetic parameters under different osmotic conditions. The results showed that *L*_p_ did not significantly differ among ejaculates, breeds, or age groups. However, there were significant differences in kinetic parameters like T10, T50, T90, and *L*_p_ between hypoosmotic (150 mOsm) and hyperosmotic (600 mOsm) conditions. While *L*_p_ shows significant variation between hypo- and hyperosmotic conditions in most groups, its utility in predicting cryopreservation outcomes such as post-thaw motility requires further investigation. This highlights the need to incorporate additional biological or molecular markers into future models to improve predictive accuracy. Moreover, incorporating goodness-of-fit indicators for *L*_p_ regressions can enhance confidence in their utility for modeling osmotic behavior and improving AI-driven predictions of cryopreservation outcomes. In the present study, although high-quality fits were achieved under both hypoosmotic and hyperosmotic conditions, model performance was notably superior under hypoosmotic stress. This was reflected by lower SSE values, higher R^2^, narrower confidence intervals for *L*_p_, and more negative AIC scores, indicating more accurate and consistent parameter estimation in hypoosmotic environments. Overall, this study demonstrates that stopped-flow fluorometry combined with nonlinear log-logistic and linear modeling provides a useful framework for characterizing sperm osmotic responses. These models provide robust quantitative tools for estimating *L*_p_ and characterizing osmotic transport kinetics in bovine sperm. Their application may facilitate future mechanistic and optimization studies of cryopreservation protocols, although the present study does not demonstrate direct improvements in post-thaw motility or cryopreservation outcomes.

## Supporting information

S1 File Supplementary tables S1–S8. This file contains supplementary tables summarizing hydraulic conductivity (*L*_p_) estimates, model fit statistics, comparisons between hypoosmotic and hyperosmotic conditions, direct associations and standardized regression analyses between *L*_p_ and post-thaw motility, sensitivity power analysis, explained variance of principal components, and PCA variable loadings.
(DOCX)

S1 Code Codes 01–03 and associated excel files. This file contains the codes and input/output Excel files used to convert fluorescence intensity to cell volume, estimate hydraulic conductivity (*L*_p_), and fit the log-logistic models.
(ZIP)

S2 Code Additional codes used for statistical analyses and figure generation. This file contains the remaining Python codes used for t-tests, age-group analyses, breed comparisons, paired comparisons, linear regression analyses, and figure generation.
(ZIP)
