# Supplementary material for: Hyper- and hypoosmotic water permeability of bovine sperm as a function of age, breed, and ejaculate number: Implications for cryopreservation
Source: PLoS One. 2026 Sep 28;21(9):e0358553. doi: 10.1371/journal.pone.0358553 (PMC13618955; doi:10.1371/journal.pone.0358553)
Supplement: Supplementary file 1 [file pone.0358553.s001.docx]

S1 Table. Summary of hydraulic conductivity (Lp) under hypoosmotic conditions (150 mOsm), estimates and model fit statistics for individual samples.

| Samples | Lp (μm/min/atm) | SSE | Standard Error | 95% CI Lower | 95% CI Upper | R^2^ | AIC |
| --- | --- | --- | --- | --- | --- | --- | --- |
| B1 | 0.41943 | 229.9288 | 0.00255537 | 0.414418831 | 0.424441764 | 0.968882 | -4324.27 |
| B2 | 0.592515 | 58.16081 | 0.001986943 | 0.588618068 | 0.59641146 | 0.989913 | -7073.38 |
| B3 | 0.403863 | 115.6165 | 0.002699475 | 0.398568498 | 0.409156657 | 0.981441 | -5699.25 |
| B4 | 0.459953 | 314.5883 | 0.005111058 | 0.449929419 | 0.469976535 | 0.935312 | -3697.28 |
| B5 | 0.44581 | 125.4272 | 0.00323238 | 0.439470946 | 0.452149318 | 0.982125 | -5536.35 |
| B6 | 0.464349 | 116.0589 | 0.004378422 | 0.455761805 | 0.472935301 | 0.978658 | -5691.61 |
| B7 | 0.430138 | 151.9508 | 0.003515913 | 0.423243081 | 0.437033555 | 0.978197 | -5152.69 |
| B8 | 0.44849 | 160.5188 | 0.004988665 | 0.438706535 | 0.458273591 | 0.978062 | -5042.98 |
| B9 | 0.418542 | 147.0358 | 0.003460899 | 0.411754603 | 0.425329295 | 0.976424 | -5218.45 |
| B10 | 0.419661 | 138.5234 | 0.003852774 | 0.41210509 | 0.427216838 | 0.979183 | -5337.73 |
| B11 | 0.555833 | 126.0487 | 0.005075582 | 0.545879211 | 0.565787181 | 0.974796 | -5526.47 |
| B12 | 0.57022 | 65.20951 | 1.8632E-05 | 0.570182999 | 0.57025608 | 0.988089 | -6844.59 |
| B13 | 0.486091 | 65.05784 | 4.08113E-05 | 0.486010593 | 0.486170668 | 0.989635 | -6849.25 |
| B14 | 0.44103 | 270.4502 | 0.00049711 | 0.440055268 | 0.442005083 | 0.966967 | -3999.63 |
| B15 | 0.3903 | 196.0445 | 0.001046769 | 0.388247286 | 0.392353033 | 0.970639 | -4643.12 |
| B16 | 0.470894 | 59.63377 | 0.003925992 | 0.463194368 | 0.478593297 | 0.990286 | -7023.36 |
| B17 | 0.405838 | 144.4258 | 0.003223583 | 0.39951572 | 0.412159588 | 0.976404 | -5254.27 |
| B18 | 0.428202 | 128.5662 | 0.00309242 | 0.422136839 | 0.434266247 | 0.98048 | -5486.92 |
| B19 | 0.474712 | 62.16847 | 0.003991077 | 0.466885155 | 0.482539367 | 0.989499 | -6940.11 |
| B20 | 0.457423 | 110.3134 | 0.002350575 | 0.452813472 | 0.462033138 | 0.98357 | -5793.15 |
| B21 | 0.396628 | 192.2226 | 3.5129E-05 | 0.396558899 | 0.396696685 | 0.970301 | -4682.5 |
| B22 | 0.42317 | 136.0201 | 0.00037137 | 0.422441792 | 0.423898417 | 0.979046 | -5374.2 |
| B23 | 0.434582 | 142.7634 | 0.000667406 | 0.433273033 | 0.435890801 | 0.978016 | -5277.43 |
| B24 | 0.439316 | 146.9296 | 0.003553194 | 0.432347895 | 0.446284598 | 0.979532 | -5219.9 |
| B25 | 0.404768 | 150.1394 | 0.000759747 | 0.403277943 | 0.4062579 | 0.977923 | -5176.68 |
| B26 | 0.348813 | 365.7918 | 6.64048E-06 | 0.348799574 | 0.34882562 | 0.947736 | -3395.68 |
| B27 | 0.450526 | 155.5257 | 5.87967E-06 | 0.450514817 | 0.450537878 | 0.970869 | -5106.18 |
| B28 | 0.398763 | 237.3887 | 0.002854936 | 0.393164274 | 0.404362198 | 0.967438 | -4260.41 |
| B29 | 0.422558 | 129.4912 | 0.001896962 | 0.418837887 | 0.426278348 | 0.977407 | -5472.58 |
| B30 | 0.379828 | 242.645 | 0.002959675 | 0.37402327 | 0.38563201 | 0.962847 | -4216.61 |
| B31 | 0.356945 | 290.2114 | 3.43308E-06 | 0.356938252 | 0.356951718 | 0.955069 | -3858.59 |
| B32 | 0.372643 | 308.3176 | 2.38316E-06 | 0.372638404 | 0.372647751 | 0.956308 | -3737.54 |
| B33 | 0.410338 | 119.7106 | 2.20495E-05 | 0.410294468 | 0.410380953 | 0.982715 | -5629.65 |
| B34 | 0.398962 | 153.2311 | 0.002212011 | 0.394623583 | 0.403299758 | 0.974732 | -5135.91 |
| B35 | 0.380928 | 322.1388 | 0.000700802 | 0.379553134 | 0.382301893 | 0.956656 | -3649.84 |
| B36 | 0.366923 | 414.7097 | 0.002620368 | 0.36178444 | 0.372062317 | 0.948359 | -3144.65 |
| B37 | 0.37828 | 289.565 | 1.36274E-06 | 0.378277145 | 0.37828249 | 0.961654 | -3863.05 |
| B38 | 0.35055 | 321.7114 | 0.002653317 | 0.345346555 | 0.35575367 | 0.956129 | -3652.5 |
| B39 | 0.409631 | 131.5071 | 0.004091676 | 0.401606384 | 0.417655177 | 0.97925 | -5441.68 |
| B40 | 0.347894 | 369.7492 | 0.002633927 | 0.34272884 | 0.3530599 | 0.9474 | -3374.16 |
| B41 | 0.459777 | 114.5895 | 0.006415447 | 0.447195652 | 0.472358979 | 0.98096 | -5717.09 |
| B42 | 0.433093 | 122.1385 | 0.002746691 | 0.427706205 | 0.43847956 | 0.97877 | -5589.49 |
| B43 | 0.381548 | 231.1572 | 0.00037177 | 0.380819223 | 0.382277418 | 0.965294 | -4313.61 |
| B44 | 0.485501 | 160.4761 | 0.002689463 | 0.480226172 | 0.49077506 | 0.969934 | -5043.51 |
| B45 | 0.400064 | 192.9404 | 0.003181028 | 0.393825175 | 0.406302132 | 0.968012 | -4675.04 |
| B46 | 0.437958 | 134.7364 | 0.003026084 | 0.432023377 | 0.443892595 | 0.978116 | -5393.16 |
| B47 | 0.425268 | 148.7514 | 0.003096162 | 0.419196418 | 0.431340503 | 0.973207 | -5195.25 |
| B48 | 0.383367 | 236.5994 | 0.002969425 | 0.377543406 | 0.389190388 | 0.965757 | -4267.07 |
| B49 | 0.396582 | 137.1829 | 0.003134395 | 0.390434596 | 0.402728641 | 0.978696 | -5357.17 |
| B50 | 0.389155 | 171.1823 | 0.003061131 | 0.383152077 | 0.395158758 | 0.97253 | -4914.35 |
| B51 | 0.388091 | 177.9507 | 0.003328997 | 0.381562636 | 0.394619972 | 0.973046 | -4836.79 |
| B52 | 0.407787 | 178.5823 | 0.00106628 | 0.405695405 | 0.409877679 | 0.974357 | -4829.71 |
| B53 | 0.400348 | 168.0429 | 0.003156095 | 0.394158852 | 0.406538012 | 0.975047 | -4951.37 |
| B54 | 0.393089 | 195.9274 | 0.003083244 | 0.387041858 | 0.399135273 | 0.969192 | -4644.32 |
| B55 | 0.365661 | 271.4524 | 0.001082383 | 0.363538555 | 0.36778399 | 0.961556 | -3992.23 |
| B56 | 0.493628 | 125.8447 | 0.003659264 | 0.486451454 | 0.500804195 | 0.976942 | -5529.71 |
| B57 | 0.396922 | 247.7866 | 0.003125865 | 0.390791629 | 0.403052217 | 0.959679 | -4174.67 |
| B58 | 0.459483 | 259.2602 | 0.003912067 | 0.451810952 | 0.467155263 | 0.944556 | -4084.14 |
| B59 | 0.478059 | 64.77722 | 0.002528776 | 0.473099709 | 0.483018332 | 0.989157 | -6857.9 |
| B60 | 0.404643 | 148.5379 | 0.002989089 | 0.398781232 | 0.410505343 | 0.977179 | -5198.12 |
| B61 | 0.440551 | 135.3316 | 0.002619665 | 0.435413588 | 0.445688707 | 0.978787 | -5384.35 |
| B62 | 0.473725 | 106.4002 | 0.005023384 | 0.463873065 | 0.483576297 | 0.984762 | -5865.39 |
| B63 | 0.387309 | 221.1774 | 0.002477432 | 0.382450706 | 0.392167944 | 0.96276 | -4401.87 |
| B64 | 0.443081 | 156.4219 | 2.21795E-05 | 0.443037107 | 0.443124101 | 0.978434 | -5094.69 |
| B65 | 0.347943 | 380.6319 | 0.002239488 | 0.343550797 | 0.352334749 | 0.934222 | -3316.14 |
| B66 | 0.449925 | 99.07899 | 0.002509253 | 0.445004027 | 0.454846077 | 0.98362 | -6007.97 |
| B67 | 0.378857 | 216.2694 | 4.72703E-06 | 0.378847396 | 0.378865937 | 0.965963 | -4446.75 |
| B68 | 0.449679 | 106.7592 | 0.003667785 | 0.442485711 | 0.456871876 | 0.982089 | -5858.65 |
| B69 | 0.401598 | 199.2243 | 0.003129959 | 0.39545954 | 0.407736186 | 0.972656 | -4610.94 |
| B70 | 0.394853 | 179.3521 | 0.003102752 | 0.388767614 | 0.400937546 | 0.971939 | -4821.1 |
| B71 | 0.366359 | 186.0629 | 0.002874403 | 0.360721492 | 0.371995773 | 0.970673 | -4747.64 |
| B72 | 0.522167 | 123.698 | 0.004542671 | 0.51325815 | 0.531075883 | 0.978395 | -5564.12 |
| B73 | 0.392358 | 279.9742 | 0.001485205 | 0.389444891 | 0.395270314 | 0.962361 | -3930.41 |
| B74 | 0.339411 | 379.5434 | 0.00271487 | 0.334086358 | 0.344734901 | 0.94114 | -3321.87 |
| B75 | 0.292433 | 691.9229 | 0.002195401 | 0.288127088 | 0.296738115 | 0.906372 | -2120.86 |
| B76 | 0.525701 | 87.74534 | 0.002471452 | 0.520854061 | 0.530547843 | 0.987128 | -6250.93 |
| B77 | 0.367322 | 278.8395 | 0.003046794 | 0.361347093 | 0.373297541 | 0.958046 | -3938.53 |
| B78 | 0.537264 | 48.36312 | 2.9125E-05 | 0.537206609 | 0.537320846 | 0.992032 | -7442.33 |
| B79 | 0.457518 | 161.7346 | 0.001965607 | 0.453663156 | 0.461372862 | 0.978231 | -5027.89 |
| B80 | 0.424661 | 134.681 | 0.006427755 | 0.412054873 | 0.437266475 | 0.976653 | -5393.99 |
| B81 | 0.460115 | 72.23992 | 0.001175782 | 0.457809256 | 0.462421031 | 0.988576 | -6639.82 |
| B82 | 0.439069 | 132.0044 | 2.49512E-05 | 0.439020433 | 0.4391183 | 0.981267 | -5434.13 |
| B83 | 0.477699 | 88.88908 | 0.003931953 | 0.469987783 | 0.485410094 | 0.98698 | -6225.03 |

S2 Table. Summary of hydraulic conductivity (Lp) under hyperosmotic conditions (600 mOsm), estimates and model fit statistics for individual Samples.

| Samples | Lp (μm/min/atm) | SSE | Standard Error | 95% CI Lower | 95% CI Upper | R² | AIC |
| --- | --- | --- | --- | --- | --- | --- | --- |
| B1 | 0.655 | 81.06367 | 0.009234606 | 0.636889148 | 0.673110069 | 0.98059 | -6409.34 |
| B2 | 0.394717 | 244.129 | 0.00485278 | 0.385199976 | 0.404234049 | 0.95052 | -4204.41 |
| B3 | 0.537415 | 427.8679 | 0.007694624 | 0.522324275 | 0.55250492 | 0.884593 | -3082.18 |
| B4 | 0.471417 | 520.8237 | 0.003700178 | 0.464160759 | 0.478673979 | 0.870149 | -2688.98 |
| B5 | 0.497141 | 192.8271 | 0.005233044 | 0.486878528 | 0.50740411 | 0.957118 | -4676.22 |
| B6 | 0.425974 | 488.5665 | 4.60483E-06 | 0.425965175 | 0.425983236 | 0.881703 | -2816.85 |
| B7 | 0.638256 | 811.2671 | 0.010670849 | 0.617328492 | 0.659182794 | 0.867445 | -1802.61 |
| B8 | 0.414977 | 137.8364 | 0.000316097 | 0.414356725 | 0.415596556 | 0.974249 | -5347.67 |
| B9 | 0.390298 | 405.912 | 0.004525805 | 0.38142253 | 0.399174107 | 0.910717 | -3187.53 |
| B10 | 0.784545 | 112.876 | 0.012128654 | 0.760758951 | 0.808331205 | 0.970396 | -5747.23 |
| B11 | 0.604023 | 528.0706 | 3.69121E-06 | 0.604015736 | 0.604030214 | 0.840959 | -2661.35 |
| B12 | 0.491572 | 169.2293 | 0.00546881 | 0.480847261 | 0.502297588 | 0.964238 | -4937.3 |
| B13 | 0.376697 | 282.8465 | 0.003092636 | 0.370631865 | 0.382762119 | 0.943589 | -3910 |
| B14 | 0.595091 | 198.6306 | 0.000245298 | 0.594609952 | 0.595572084 | 0.950971 | -4616.91 |
| B15 | 0.468623 | 206.9806 | 0.006596315 | 0.455686604 | 0.481559349 | 0.955662 | -4534.55 |
| B16 | 0.686721 | 181.7446 | 0.010403371 | 0.666318271 | 0.707123444 | 0.951525 | -4794.6 |
| B17 | 0.521101 | 506.7742 | 0.007820164 | 0.505764715 | 0.536437766 | 0.864434 | -2743.67 |
| B18 | 0.701346 | 178.5169 | 0.000572099 | 0.70022356 | 0.702467504 | 0.95238 | -4830.44 |
| B19 | 0.548661 | 120.8473 | 0.00577363 | 0.537338187 | 0.559984112 | 0.972468 | -5610.75 |
| B20 | 0.620427 | 80.64813 | 0.007297593 | 0.606114929 | 0.6347383 | 0.981461 | -6419.61 |
| B21 | 0.522759 | 220.1031 | 0.006971853 | 0.509086032 | 0.536431749 | 0.950302 | -4411.61 |
| B22 | 0.427229 | 482.9114 | 0.007232311 | 0.413045137 | 0.44141245 | 0.88511 | -2840.14 |
| B23 | 0.629891 | 101.0632 | 0.008831506 | 0.61257082 | 0.64721066 | 0.975025 | -5968.31 |
| B24 | 0.579323 | 129.0497 | 5.04467E-05 | 0.579224343 | 0.57942221 | 0.969327 | -5479.41 |
| B25 | 0.574493 | 127.54 | 0.007708441 | 0.559375338 | 0.589610178 | 0.970865 | -5502.95 |
| B26 | 0.542982 | 148.0604 | 0.009910458 | 0.523545848 | 0.562417665 | 0.965848 | -5204.56 |
| B27 | 0.639427 | 153.3805 | 7.65935E-06 | 0.639412159 | 0.639442202 | 0.963386 | -5133.96 |
| B28 | 0.489691 | 141.2931 | 0.00601577 | 0.477893382 | 0.501489054 | 0.97111 | -5298.13 |
| B29 | 0.34568 | 415.5802 | 0.001548076 | 0.342643643 | 0.348715665 | 0.916537 | -3140.45 |
| B30 | 0.47895 | 205.7253 | 0.004524027 | 0.470077581 | 0.487822183 | 0.955321 | -4546.72 |
| B31 | 0.481025 | 381.4696 | 0.005946508 | 0.469363485 | 0.492687492 | 0.908337 | -3311.74 |
| B32 | 0.492633 | 309.3268 | 0.007338334 | 0.47824187 | 0.507025039 | 0.928452 | -3731.01 |
| B33 | 0.500496 | 214.4053 | 0.011420316 | 0.478098906 | 0.522892843 | 0.951873 | -4464.07 |
| B34 | 0.530122 | 402.6678 | 0.007404936 | 0.515599809 | 0.544644212 | 0.895964 | -3203.58 |
| B35 | 0.613058 | 265.0432 | 0.009557632 | 0.594314476 | 0.631802404 | 0.928103 | -4040.02 |
| B36 | 0.513572 | 158.1117 | 0.002849905 | 0.507982474 | 0.519160666 | 0.966069 | -5073.2 |
| B37 | 0.528928 | 301.1709 | 0.008396069 | 0.512462238 | 0.545394162 | 0.925323 | -3784.45 |
| B38 | 0.683555 | 98.97244 | 1.44206E-05 | 0.683527214 | 0.683583776 | 0.975177 | -6010.12 |
| B39 | 0.77485 | 177.2453 | 0.00014344 | 0.774568768 | 0.775131384 | 0.956629 | -4844.74 |
| B40 | 0.539676 | 303.5609 | 0.007381854 | 0.52519937 | 0.554153237 | 0.923206 | -3768.64 |
| B41 | 0.466154 | 445.3696 | 0.009160211 | 0.448189733 | 0.484118853 | 0.890793 | -3002 |
| B42 | 0.660397 | 431.8876 | 0.007315026 | 0.646050697 | 0.674742446 | 0.86521 | -3063.47 |
| B43 | 0.500603 | 251.0396 | 0.006427987 | 0.487996515 | 0.513209028 | 0.944424 | -4148.58 |
| B44 | 0.626318 | 523.768 | 0.016170523 | 0.594605596 | 0.658031283 | 0.836829 | -2677.71 |
| B45 | 0.757613 | 59.44388 | 0.005312416 | 0.7471948 | 0.768031703 | 0.98384 | -7029.74 |
| B46 | 0.716582 | 124.4575 | 0.007000749 | 0.702852122 | 0.73031118 | 0.965692 | -5551.88 |
| B47 | 0.710757 | 79.06288 | 0.007797224 | 0.695465182 | 0.726048256 | 0.979729 | -6459.32 |
| B48 | 0.48208 | 222.0571 | 3.45404E-06 | 0.482073403 | 0.48208695 | 0.949798 | -4393.94 |
| B49 | 0.412622 | 584.071 | 4.0792E-05 | 0.412541862 | 0.412701861 | 0.859549 | -2459.76 |
| B50 | 0.451851 | 478.0495 | 1.67631E-06 | 0.451847343 | 0.451853918 | 0.885167 | -2860.38 |
| B51 | 0.831237 | 108.1406 | 0.000202947 | 0.830838985 | 0.831635006 | 0.97053 | -5832.94 |
| B52 | 0.58934 | 188.3539 | 4.16201E-05 | 0.589258049 | 0.589421296 | 0.953281 | -4723.16 |
| B53 | 0.647453 | 131.7437 | 0.009133695 | 0.629540015 | 0.665365131 | 0.968384 | -5438.09 |
| B54 | 0.592971 | 235.6103 | 0.032236799 | 0.529750035 | 0.656192522 | 0.943679 | -4275.45 |
| B55 | 0.777428 | 108.3672 | 0.022496616 | 0.733308324 | 0.821546864 | 0.971975 | -5828.75 |
| B56 | 0.877676 | 163.9408 | 0.033443851 | 0.812087693 | 0.943264606 | 0.960301 | -5000.79 |
| B57 | 0.498377 | 254.708 | 0.004416327 | 0.489715594 | 0.507037764 | 0.942571 | -4119.57 |
| B58 | 0.572326 | 442.2455 | 0.008481692 | 0.555692051 | 0.588959815 | 0.892344 | -3016.07 |
| B59 | 0.413752 | 395.3939 | 0.006474048 | 0.401055218 | 0.426448395 | 0.912778 | -3240.04 |
| B60 | 0.627802 | 92.32777 | 0.005668207 | 0.61668625 | 0.638918672 | 0.979279 | -6149.12 |
| B61 | 0.416119 | 426.5246 | 0.004832536 | 0.406642136 | 0.425596806 | 0.901689 | -3088.46 |
| B62 | 0.450636 | 261.1283 | 0.008058567 | 0.434831577 | 0.466439718 | 0.943228 | -4069.78 |
| B63 | 0.920862 | 344.5589 | 0.005229453 | 0.910606491 | 0.931117988 | 0.920887 | -3515.27 |
| B64 | 0.404689 | 290.0691 | 1.76291E-06 | 0.404685903 | 0.404692817 | 0.939194 | -3859.57 |
| B65 | 0.416679 | 390.3826 | 0.004542503 | 0.407770202 | 0.425587275 | 0.91478 | -3265.55 |
| B66 | 0.571703 | 210.0732 | 0.007276395 | 0.557433274 | 0.585973498 | 0.94846 | -4504.89 |
| B67 | 0.739393 | 42.60616 | 1.51781E-05 | 0.7393632 | 0.739422733 | 0.989316 | -7695.81 |
| B68 | 0.673961 | 69.46103 | 0.002207123 | 0.669632374 | 0.67828938 | 0.982335 | -6718.27 |
| B69 | 0.463577 | 743.0832 | 0.01164376 | 0.440741883 | 0.486412235 | 0.78879 | -1978.19 |
| B70 | 1.077689 | 549.4543 | 0.018907693 | 1.040608192 | 1.114769891 | 0.865372 | -2581.95 |
| B71 | 0.434909 | 444.0652 | 9.70197E-06 | 0.434889497 | 0.434927551 | 0.893357 | -3007.86 |
| B72 | 0.372739 | 678.9131 | 0.004336965 | 0.364233976 | 0.381244866 | 0.841157 | -2158.82 |
| B73 | 0.403475 | 252.5058 | 0.00462062 | 0.394413744 | 0.412537216 | 0.948352 | -4136.94 |
| B74 | 0.655286 | 184.3027 | 0.008039456 | 0.639519237 | 0.671052419 | 0.951345 | -4766.65 |
| B75 | 0.605902 | 115.9049 | 0.015989828 | 0.574543729 | 0.637260678 | 0.973031 | -5694.27 |
| B76 | 0.927809 | 259.2255 | 0.026174406 | 0.876476707 | 0.979140653 | 0.937445 | -4084.41 |
| B77 | 0.683384 | 88.38944 | 0.009483267 | 0.664785545 | 0.701981789 | 0.978346 | -6236.3 |
| B78 | 0.420073 | 456.7825 | 8.67649E-06 | 0.42005592 | 0.420089952 | 0.893033 | -2951.39 |
| B79 | 0.42931 | 226.0765 | 0.000831839 | 0.427678623 | 0.430941346 | 0.953255 | -4358.06 |
| B80 | 0.952816 | 90.09201 | 0.015171953 | 0.923061112 | 0.982570104 | 0.973663 | -6198.14 |
| B81 | 0.584528 | 265.576 | 0.013521595 | 0.558010272 | 0.611046062 | 0.929861 | -4036 |
| B82 | 0.591707 | 500.0736 | 0.014422066 | 0.56342316 | 0.619990872 | 0.853707 | -2770.29 |
| B83 | 0.531045 | 403.9795 | 0.011365825 | 0.508754553 | 0.55333476 | 0.893946 | -3197.08 |

S3 Table. Sample-level comparison of hydraulic conductivity (Lp) between hypoosmotic (150 mOsm) and hyperosmotic (600 mOsm) conditions using two-sided Z-tests. Breed, age, total motility, and progressive motility are included for each bull.

| Samples | Breed | Z | Two-tail p | Significant at 0.05 | Direction | Age (months) | Total motility (%) | Progressive motility (%) |
| --- | --- | --- | --- | --- | --- | --- | --- | --- |
| B1 | Holstein | 25 | 2.E-133 | Yes | Lp600 > Lp150 | 17 | 72.67 | 53.58 |
| B2 | Holstein | -38 | 0.E+00 | Yes | Lp600 < Lp150 | 47 | 76.24 | 52.71 |
| B3 | Holstein | 16 | 3.E-60 | Yes | Lp600 > Lp150 | 28 | 58.54 | 42.35 |
| B4 | Holstein | 2 | 7.E-02 | No | Lp600 > Lp150 | 35 | 68.25 | 50.79 |
| B5 | Holstein | 8 | 7.E-17 | Yes | Lp600 > Lp150 | 69 | 70.82 | 54.96 |
| B6 | Holstein | -9 | 2.E-18 | Yes | Lp600 < Lp150 | 24 | 63.64 | 49.72 |
| B7 | Holstein | 19 | 1.E-76 | Yes | Lp600 > Lp150 | 55 | 75.75 | 60.78 |
| B8 | Holstein | -7 | 2.E-11 | Yes | Lp600 < Lp150 | 19 | 71.76 | 57.51 |
| B9 | Holstein | -5 | 7.E-07 | Yes | Lp600 < Lp150 | 19 | 68.61 | 48.23 |
| B10 | Holstein | 29 | 8.E-181 | Yes | Lp600 > Lp150 | 22 | 65.26 | 51.12 |
| B11 | Holstein | 9 | 2.E-21 | Yes | Lp600 > Lp150 | 14 | 57.18 | 42.82 |
| B12 | Holstein | -14 | 7.E-47 | Yes | Lp600 < Lp150 | 12 | 69.73 | 47.76 |
| B13 | Holstein | -35 | 5.E-274 | Yes | Lp600 < Lp150 | 22 | 67.82 | 45.62 |
| B14 | Holstein | 278 | 0.E+00 | Yes | Lp600 > Lp150 | 16 | 77.07 | 58.63 |
| B15 | Holstein | 12 | 9.E-32 | Yes | Lp600 > Lp150 | 28 | 70.81 | 61.74 |
| B16 | Holstein | 19 | 6.E-84 | Yes | Lp600 > Lp150 | 44 | 84.34 | 66.16 |
| B17 | Holstein | 14 | 3.E-42 | Yes | Lp600 > Lp150 | 17 | 76.50 | 56.45 |
| B18 | Holstein | 87 | 0.E+00 | Yes | Lp600 > Lp150 | 31 | 57.01 | 40.50 |
| B19 | Holstein | 11 | 6.E-26 | Yes | Lp600 > Lp150 | 27 | 66.01 | 49.17 |
| B20 | Holstein | 21 | 3.E-100 | Yes | Lp600 > Lp150 | 23 | 65.46 | 50.42 |
| B21 | Holstein | 18 | 4.E-73 | Yes | Lp600 > Lp150 | 26 | 77.45 | 53.08 |
| B22 | Holstein | 1 | 6.E-01 | No | Lp600 > Lp150 | 13 | 56.80 | 42.31 |
| B23 | Holstein | 22 | 9.E-108 | Yes | Lp600 > Lp150 | 27 | 68.47 | 45.05 |
| B24 | Holstein | 39 | 0.E+00 | Yes | Lp600 > Lp150 | 49 | 72.46 | 49.40 |
| B25 | Holstein | 22 | 2.E-106 | Yes | Lp600 > Lp150 | 20 | 60.84 | 43.98 |
| B26 | Holstein | 20 | 2.E-85 | Yes | Lp600 > Lp150 | 44 | 56.68 | 42.35 |
| B27 | Holstein | 19563 | 0.E+00 | Yes | Lp600 > Lp150 | 32 | 28.70 | 18.75 |
| B28 | Holstein | 14 | 2.E-42 | Yes | Lp600 > Lp150 | 50 | 69.36 | 54.41 |
| B29 | Holstein | -31 | 2.E-216 | Yes | Lp600 < Lp150 | 32 | 52.32 | 40.60 |
| B30 | Holstein | 18 | 4.E-75 | Yes | Lp600 > Lp150 | 88 | 72.17 | 51.88 |
| B31 | Holstein | 21 | 1.E-96 | Yes | Lp600 > Lp150 | 71 | 60.56 | 44.17 |
| B32 | Holstein | 16 | 4.E-60 | Yes | Lp600 > Lp150 | 38 | 79.03 | 57.14 |
| B33 | Holstein | 8 | 3.E-15 | Yes | Lp600 > Lp150 | 19 | 55.11 | 38.44 |
| B34 | Holstein | 17 | 1.E-64 | Yes | Lp600 > Lp150 | 35 | 64.21 | 44.21 |
| B35 | Holstein | 24 | 1.E-129 | Yes | Lp600 > Lp150 | 21 | 83.19 | 59.61 |
| B36 | Holstein | 38 | 0.E+00 | Yes | Lp600 > Lp150 | 51 | 67.99 | 51.32 |
| B37 | Holstein | 18 | 5.E-72 | Yes | Lp600 > Lp150 | 16 | 72.47 | 48.24 |
| B38 | Holstein | 126 | 0.E+00 | Yes | Lp600 > Lp150 | 46 | 78.71 | 64.43 |
| B39 | Holstein | 89 | 0.E+00 | Yes | Lp600 > Lp150 | 46 | 62.69 | 50.76 |
| B40 | Holstein | 24 | 3.E-132 | Yes | Lp600 > Lp150 | 26 | 83.33 | 52.38 |
| B41 | Holstein | 1 | 6.E-01 | No | Lp600 > Lp150 | 18 | 62.24 | 39.45 |
| B42 | Holstein | 29 | 5.E-186 | Yes | Lp600 > Lp150 | 32 | 69.94 | 53.80 |
| B43 | Holstein | 18 | 2.E-76 | Yes | Lp600 > Lp150 | 46 | 79.88 | 63.47 |
| B44 | Holstein | 9 | 9.E-18 | Yes | Lp600 > Lp150 | 32 | 74.17 | 54.05 |
| B45 | Holstein | 58 | 0.E+00 | Yes | Lp600 > Lp150 | 46 | 57.47 | 42.21 |
| B46 | Holstein | 37 | 3.E-292 | Yes | Lp600 > Lp150 | 18 | 63.84 | 42.14 |
| B47 | Holstein | 34 | 8.E-254 | Yes | Lp600 > Lp150 | 22 | 47.62 | 29.89 |
| B48 | Holstein | 33 | 3.E-242 | Yes | Lp600 > Lp150 | 25 | 77.51 | 62.35 |
| B49 | Holstein | 5 | 3.E-07 | Yes | Lp600 > Lp150 | 14 | 62.87 | 44.15 |
| B50 | Holstein | 20 | 3.E-93 | Yes | Lp600 > Lp150 | 20 | 58.58 | 36.15 |
| B51 | Holstein | 133 | 0.E+00 | Yes | Lp600 > Lp150 | 46 | 60.00 | 42.57 |
| B52 | Holstein | 170 | 0.E+00 | Yes | Lp600 > Lp150 | 32 | 76.01 | 57.32 |
| B53 | Holstein | 26 | 3.E-144 | Yes | Lp600 > Lp150 | 13 | 66.74 | 51.99 |
| B54 | Holstein | 6 | 7.E-10 | Yes | Lp600 > Lp150 | 38 | 72.24 | 47.83 |
| B55 | Holstein | 18 | 1.E-74 | Yes | Lp600 > Lp150 | 24 | 77.12 | 55.77 |
| B56 | Jersey | 11 | 4.E-30 | Yes | Lp600 > Lp150 | 14 | 65.58 | 49.07 |
| B57 | Jersey | 19 | 2.E-78 | Yes | Lp600 > Lp150 | 57 | 70.15 | 55.97 |
| B58 | Jersey | 12 | 1.E-33 | Yes | Lp600 > Lp150 | 13 | 57.29 | 38.28 |
| B59 | Jersey | -9 | 2.E-20 | Yes | Lp600 < Lp150 | 16 | 50.25 | 37.75 |
| B60 | Charolais | 35 | 1.E-265 | Yes | Lp600 > Lp150 | 24 | 60.99 | 45.86 |
| B61 | Angus | -4 | 9.E-06 | Yes | Lp600 < Lp150 | 23 | 57.86 | 42.64 |
| B62 | Jersey | -2 | 2.E-02 | Yes | Lp600 < Lp150 | 81 | 25.12 | 17.66 |
| B63 | Jersey | 92 | 0.E+00 | Yes | Lp600 > Lp150 | 16 | 68.01 | 50.00 |
| B64 | Jersey | -1726 | 0.E+00 | Yes | Lp600 < Lp150 | 81 | 76.07 | 60.04 |
| B65 | Jersey | 14 | 6.E-42 | Yes | Lp600 > Lp150 | 14 | 65.78 | 53.40 |
| B66 | Jersey | 16 | 2.E-56 | Yes | Lp600 > Lp150 | 16 | 49.93 | 33.26 |
| B67 | Charolais | 22679 | 0.E+00 | Yes | Lp600 > Lp150 | 25 | 60.19 | 45.86 |
| B68 | Jersey | 52 | 0.E+00 | Yes | Lp600 > Lp150 | 14 | 67.06 | 49.26 |
| B69 | Holstein | 5 | 3.E-07 | Yes | Lp600 > Lp150 | 32 | 50.00 | 39.14 |
| B70 | Holstein | 36 | 4.E-278 | Yes | Lp600 > Lp150 | 16 | 78.54 | 57.58 |
| B71 | Holstein | 24 | 1.E-125 | Yes | Lp600 > Lp150 | 29 | 59.71 | 43.82 |
| B72 | Holstein | -24 | 4.E-125 | Yes | Lp600 < Lp150 | 116 | 64.76 | 44.93 |
| B73 | Jersey | 2 | 2.E-02 | Yes | Lp600 > Lp150 | 20 | 60.27 | 47.30 |
| B74 | Holstein | 37 | 3.E-303 | Yes | Lp600 > Lp150 | 14 | 75.44 | 61.70 |
| B75 | Holstein | 19 | 5.E-84 | Yes | Lp600 > Lp150 | 21 | 77.85 | 47.03 |
| B76 | Jersey | 15 | 8.E-53 | Yes | Lp600 > Lp150 | 40 | 62.65 | 49.17 |
| B77 | Holstein | 32 | 6.E-221 | Yes | Lp600 > Lp150 | 22 | 80.00 | 63.75 |
| B78 | Jersey | -3856 | 0.E+00 | Yes | Lp600 < Lp150 | 46 | 51.05 | 35.71 |
| B79 | Holstein | -13 | 7.E-40 | Yes | Lp600 < Lp150 | 31 | 74.63 | 55.67 |
| B80 | Holstein | 32 | 2.E-225 | Yes | Lp600 > Lp150 | 33 | 67.12 | 53.42 |
| B81 | Holstein | 9 | 5.E-20 | Yes | Lp600 > Lp150 | 22 | 80.04 | 62.87 |
| B82 | Holstein | 11 | 4.E-26 | Yes | Lp600 > Lp150 | 47 | 45.83 | 30.51 |
| B83 | Holstein | 4 | 9.E-06 | Yes | Lp600 > Lp150 | 18 | 70.32 | 45.37 |

S4 Table. Pairwise associations of hydraulic conductivity with post thaw total and progressive motility.

| Outcome | Predictor | N | Pearson r | 95% CI lower | 95% CI upper | Pearson p | Spearman rho | Spearman p | Pearson FDR-adjusted p | Spearman FDR-adjusted p |
| --- | --- | --- | --- | --- | --- | --- | --- | --- | --- | --- |
| TM | Lp150 | 83 | -0.215 | -0.411 | 0.001 | 0.051 | -0.235 | 0.033 | 0.103 | 0.077 |
| TM | Lp600 | 83 | 0.113 | -0.105 | 0.321 | 0.308 | 0.127 | 0.254 | 0.308 | 0.254 |
| PM | Lp150 | 83 | -0.217 | -0.414 | -0.002 | 0.049 | -0.228 | 0.038 | 0.103 | 0.077 |
| PM | Lp600 | 83 | 0.132 | -0.086 | 0.338 | 0.233 | 0.154 | 0.164 | 0.308 | 0.218 |

Pearson and Spearman correlation coefficients were calculated using 83 separately collected semen samples. The 95% confidence intervals are shown for Pearson correlations. P values were adjusted for four comparisons using the false discovery rate method. TM, total motility; PM, progressive motility; Lp150, hydraulic conductivity after mixing with the 150 mOsm medium, corresponding to 220 mOsm final osmolality; Lp600, hydraulic conductivity after mixing with the 600 mOsm medium, corresponding to 445 mOsm final osmolality.

S5 Table. Standardized regression analyses of hydraulic conductivity and post thaw motility.

| Outcome | Predictor | N observations | Standardized beta | SE | 95% CI lower | 95% CI upper | p value |
| --- | --- | --- | --- | --- | --- | --- | --- |
| TM | Lp150 | 83 | -0.215 | 0.109 | -0.431 | 0.001 | 0.051 |
| TM | Lp600 | 83 | 0.113 | 0.110 | -0.106 | 0.333 | 0.308 |
| PM | Lp150 | 83 | -0.217 | 0.108 | -0.433 | -0.001 | 0.049 |
| PM | Lp600 | 83 | 0.132 | 0.110 | -0.087 | 0.352 | 0.233 |

Standardized ordinary least squares regression models were fitted using 83 separately collected semen samples. Standardized beta represents the change in the outcome, expressed in standard deviation units, for a one standard deviation increase in hydraulic conductivity. TM, total motility; PM, progressive motility.

S6 Table. Sensitivity power analysis for two sided Pearson correlation tests.

| Outcome | Predictor | N | Alpha | Target power | Minimum detectable \|r\| | Power for \|r\| = 0.20 | Power for \|r\| = 0.30 | Power for \|r\| = 0.40 |
| --- | --- | --- | --- | --- | --- | --- | --- | --- |
| TM | Lp150 | 83 | 0.050 | 0.800 | 0.303 | 0.442 | 0.791 | 0.966 |
| TM | Lp600 | 83 | 0.050 | 0.800 | 0.303 | 0.442 | 0.791 | 0.966 |
| PM | Lp150 | 83 | 0.050 | 0.800 | 0.303 | 0.442 | 0.791 | 0.966 |
| PM | Lp600 | 83 | 0.050 | 0.800 | 0.303 | 0.442 | 0.791 | 0.966 |

The minimum detectable correlation represents the smallest absolute Pearson correlation expected to be detected with 80% power using a two sided test at α = 0.05 and n = 83. The power values shown for absolute correlations of 0.20, 0.30, and 0.40 are based on the same assumptions.

S7 Table. Variance explained by the principal components included in the multivariate analysis.

| Principal component | Explained variance (%) | Cumulative variance (%) |
| --- | --- | --- |
| PC1 | 34.830 | 34.830 |
| PC2 | 22.264 | 57.095 |
| PC3 | 19.746 | 76.841 |
| PC4 | 11.668 | 88.509 |
| PC5 | 6.117 | 94.625 |
| PC6 | 2.297 | 96.923 |

S8 Table. Variable loadings for the first two principal components.

| Variable | PC1 | PC2 |
| --- | --- | --- |
| TM | -0.142 | 0.050 |
| PM | -0.142 | 0.058 |
| T10 M150 | -0.370 | -0.155 |
| T50 M150 | -0.398 | -0.156 |
| T90 M150 | -0.218 | -0.056 |
| T10 M600 | -0.083 | 0.281 |
| T50 M600 | 0.110 | -0.290 |
| T90 M600 | 0.153 | -0.418 |
| 1/T10 M150 | 0.373 | 0.148 |
| 1/T50 M150 | 0.400 | 0.152 |
| 1/T90 M150 | 0.229 | 0.062 |
| 1/T10 M600 | 0.110 | -0.192 |
| 1/T50 M600 | -0.060 | 0.275 |
| 1/T90 M600 | -0.155 | 0.463 |
| Lp150 | 0.398 | 0.136 |
| Lp600 | -0.156 | 0.459 |

All variables were standardized before principal component analysis. Larger absolute loading values indicate a greater contribution to the corresponding principal component. TM, total motility; PM, progressive motility; T10, T50, and T90, times required to achieve 10%, 50%, and 90% of the projected volume response; Lp, hydraulic conductivity.
