# Supplementary material for: Hyper- and hypoosmotic water permeability of bovine sperm as a function of age, breed, and ejaculate number: Implications for cryopreservation
Source: PLoS One. 2026 Sep 28;21(9):e0358553. doi: 10.1371/journal.pone.0358553 (PMC13618955; doi:10.1371/journal.pone.0358553)
Supplement: Supplementary file 2 [file pone.0358553.s005.docx]

Response to the Academic Editor and Reviewers

**Manuscript ID: PONE-D-26-30013**
Title: Hyper- and hypoosmotic water permeability of bovine sperm as a function of age, breed, and ejaculate number: implications for cryopreservation

We thank the Academic Editor and both reviewers for their thoughtful and constructive comments. We have carefully revised the manuscript and Supporting Information. Reviewer comments are reproduced below in italic text, followed by our responses. Where a suggested reference was not included, we provide a scientific rationale.

# Academic Editor

**Comment:** It seems essential to include a clear write-up and sample size, power analysis to complete with justification.

**Response:** We thank the Academic Editor for highlighting the need to clarify sample size and statistical power. The study included 83 separately collected and experimentally processed semen samples obtained from 60 bulls. Each sample was treated as an observational unit because each had its own osmotic-response and post-thaw motility measurements. We added direct Pearson and Spearman correlation analyses, standardized ordinary least squares regression analyses, and a sensitivity power analysis for the four primary *L*_p_–motility associations. With *n* = 83 and a two-sided α of 0.05, the minimum detectable absolute Pearson correlation at 80% power was approximately 0.303. Power was 44.2% for |r| = 0.20, 79.1% for |r| = 0.30, and 96.6% for |r| = 0.40. The revised manuscript now states that moderate to strong associations are not supported, while weak associations cannot be excluded. Complete results are provided in Tables S4–S8.

# Journal Requirements

**Comment:** Please provide an amended Funding Statement declaring all sources of support and include the statement: “There was no additional external funding received for this study.”

**Response:** The Funding Statement has been amended in the cover letter as follows: “This study was supported by the Natural Sciences and Engineering Research Council of Canada through grants RGPIN-2023-04007 and ALLRP 585227-23 awarded to James D. Benson. There was no additional external funding received for this study.”

**Comment:** Please state what role the funders took in the study.

**Response:** The following Role of the Funder statement has been included in the cover letter: “The funders had no role in study design, data collection and analysis, decision to publish, or preparation of the manuscript.”

**Comment:** Please remove funding-related text from the Acknowledgments section.

**Response:** Funding-related text has been removed from the manuscript Acknowledgments. Funding information is now provided only in the Funding Statement in the cover letter and online submission form.

**Comment:** The ethics statement should only appear in the Methods section.

**Response:** The ethics statement is retained in the Bulls and semen collection subsection of Materials and methods and has been removed from other sections of the manuscript.

**Comment:** Please evaluate reviewer-recommended references and cite them only when relevant.

**Response:** Each recommended publication was reviewed individually. Relevant references were incorporated where they directly supported the revised discussion of oxidative stress, membrane stability, ejaculate variability, and cryopreservation. One recommended mouse lead-toxicity study was not cited because it did not directly address bovine sperm cryopreservation, osmotic water transport, hydraulic conductivity, breed, age, or ejaculate effects.

# Reviewer 1

## Major comments

**Comment:** The abstract effectively summarizes key findings but overstates the predictive utility of log-logistic and linear models for cryopreservation refinements without sufficient supporting data on improved outcomes.

**Response:** We agree and revised the final sentences of the Abstract. The revised text states that the models provide reliable approaches for estimating *L*_p_ and a quantitative framework for future mechanistic studies, while explicitly noting that *L*_p_ was not a robust predictor of post-thaw motility under the freezing conditions examined. We no longer imply that the present study directly improved cryopreservation outcomes.

**Comment:** Clarify that the lack of correlation applies specifically to post-thaw motility under the study's freezing protocol.

**Response:** The Abstract, Results, Discussion, and Conclusion now explicitly state that the absence of a robust association applies to post-thaw total and progressive motility under the freezing protocol used in this study. We also clarified that weak associations cannot be excluded.

**Comment:** The transition to water permeability's role could better acknowledge membrane integrity factors during freezing. Include the recommended caffeic acid study.

**Response:** We revised the Introduction to emphasize that post-thaw sperm quality reflects multiple interacting forms of cryoinjury, including oxidative stress, lipid peroxidation, mitochondrial dysfunction, DNA damage, and loss of plasma membrane integrity. The recommended caffeic acid study was reviewed and cited as evidence that antioxidant balance and membrane preservation can substantially influence post-thaw sperm performance in buffalo bull semen.

**Comment:** Expand on why larger, multifactorial stopped-flow studies are needed and include the recommended L-proline and fulvic acid study.

**Response:** We expanded the Introduction to explain that the earlier stopped-flow work examined only seven Holstein donor bulls of unspecified age and did not directly evaluate the association between water permeability and post-thaw motility across ejaculates, breeds, and age groups. The recommended L-proline and fulvic acid study was cited as supporting evidence that cryopreservation outcomes vary with membrane-protective and antioxidant interventions, further justifying a broader multifactorial design.

**Comment:** Justification for pooling ejaculates after permeability testing requires a clearer rationale for potential bias. Include the recommended glutathione supplementation study.

**Response:** We clarified the experimental design in the Bulls and semen collection subsection. For 15 paired collection sessions, Ejaculate 1 and Ejaculate 2 were retained separately for permeability measurements and were also cryopreserved and evaluated separately for post-thaw motility. Additional aliquots were pooled and cryopreserved as a distinct sample type to reproduce the routine commercial workflow. All individual and pooled aliquots were processed under identical cryopreservation conditions to minimize processing-related bias. The manuscript now notes that pooling may average ejaculate-specific differences and therefore was not substituted for the individual measurements. The recommended glutathione study was cited to support the importance of standardizing the biochemical freezing environment.

**Comment:** The choice of a 20 s recording period for hypoosmotic conditions requires stronger justification or sensitivity analysis. Include the recommended bisphenol-S study.

**Response:** We chose the stronger-justification option requested by the reviewer rather than adding a new sensitivity analysis. The Methods now explain that the 150 mOsm treatment was intended to characterize the dynamic early osmotic swelling response, not equilibrium cell volume. Preliminary recordings extending to 120 s showed continued swelling without a stable plateau. A longer recording would therefore not have yielded a confirmed equilibrium endpoint and would have prolonged severe hypoosmotic exposure, increasing the likelihood of secondary membrane and regulatory-volume responses unrelated to the initial passive water-transport process. The manuscript now consistently describes the 20 s endpoint as an operational dynamic-response measure. The recommended bisphenol-S study was cited only as complementary evidence that reproductive cells are sensitive to membrane-associated and oxidative stress, not as direct evidence for the stopped-flow recording duration.

**Comment:** The statistical section could specify power analysis for detecting correlations, given the negative result.

**Response:** A sensitivity power analysis was added. With *n* = 83 and α = 0.05, the study had 80% power to detect an absolute Pearson correlation of approximately 0.303. Power was 44.2% for |r| = 0.20, 79.1% for |r| = 0.30, and 96.6% for |r| = 0.40. These results are described in the Statistical analysis, Results, and Discussion sections and reported in Table S6.

**Comment:** The lack of Lp–motility correlation requires deeper exploration of alternative factors such as oxidative stress. Include the glutathione and trehalose study.

**Response:** We expanded the Results interpretation and Discussion to explain that osmotic water transport alone does not account for variability in post-thaw motility. The revised text discusses oxidative stress, lipid peroxidation, mitochondrial dysfunction, plasma membrane integrity, and extender composition as alternative or interacting determinants. The recommended glutathione and trehalose study was cited as complementary evidence that antioxidant balance and membrane protection can modify sperm quality independently of hydraulic conductivity. We explicitly note that this study concerned liquid storage at 5 °C rather than freeze-thaw cryopreservation.

**Comment:** Age and breed effects on motility are well-noted, but mechanistic links to Lp could be strengthened. Include the recommended carob extract mouse study.

**Response:** We considered the recommended study carefully. Although it provides useful evidence regarding oxidative stress and sperm function in a mouse model of lead-induced reproductive toxicity, its experimental context differs substantially from the present study and does not address bovine sperm cryopreservation, hydraulic conductivity, osmotic transport, breed, age, or ejaculate effects. We therefore did not cite this reference to avoid implying a direct mechanistic connection that was not investigated. The Discussion nevertheless acknowledges that age- and breed-related variation in post-thaw function may reflect broader differences in membrane composition, oxidative status, and mitochondrial function in addition to *L*_p_.

**Comment:** Clinical/translational implications for AI centers could more explicitly address why Lp may still be useful indirectly, for example through membrane fluidity.

**Response:** The revised Discussion clarifies that *L*_p_ may provide indirect mechanistic information about membrane transport behavior, membrane fluidity, osmotic tolerance, and cryoprotectant equilibration. We describe *L*_p_ as a process-development and characterization parameter rather than a stand-alone predictor of semen quality or fertility.

**Comment:** Some boxplots, for example Fig. 7, would benefit from percentage annotations.

**Response:** We considered adding percentage annotations directly to Fig. 7. We respectfully retained the original figure format because it already contains multiple group comparisons, individual observations, and significance indicators. Adding percentage values would substantially increase visual complexity and reduce readability. The principal quantitative differences are reported in the Results, while the figure is intended to display distributions and statistical comparisons rather than every numerical value.

**Comment:** PCA and regression results should better address why motility parameters cluster near the origin.

**Response:** The Results now report the numerical PCA loadings for total and progressive motility and explain that their small loadings on PC1 and PC2 indicate a limited contribution to the variance represented by those axes. We also clarified that proximity to the origin is descriptive and is not a formal test of absence of association. Direct correlation and standardized regression analyses were therefore added as the formal assessments. Full PCA explained variance and loadings are provided in Tables S7 and S8.

## Minor comments

**Comment:** The title/short title should use parallel phrasing.

**Response:** The title was revised to: “Hyper- and hypoosmotic water permeability of bovine sperm as a function of age, breed, and ejaculate number: implications for cryopreservation.”

**Comment:** Ensure consistent use of hypoosmotic versus hypotonic.

**Response:** Terminology was standardized throughout the revised manuscript to use hypoosmotic and hyperosmotic for the experimental media and conditions.

**Comment:** Some Introduction sentences are lengthy; split the Gilmore/Agca discussion.

**Response:** Long sentences in the Introduction were divided and revised for clarity and logical flow.

**Comment:** Correct “Chaviero” to “Chaveiro.”

**Response:** The spelling has been corrected throughout the manuscript.

**Comment:** Clarify CFDA loading concentration and preparation.

**Response:** The CFDA loading concentration was verified and corrected to 5 µM. The loading and washing procedure is now stated clearly.

**Comment:** Clarify the Boyle–van’t Hoff interpretation and the non-equilibrium hypoosmotic endpoint.

**Response:** The manuscript now states that the hypoosmotic endpoint is an operational dynamic-response estimate and that the three-point Boyle–van’t Hoff analysis was used to assess consistency with the expected osmotic trend, not to prove complete equilibrium or estimate total osmotically inactive volume.

**Comment:** Clarify T10, T50, and T90 definitions.

**Response:** The Methods clarify that these are model-derived times to 10%, 50%, and 90% of the projected response. The limitations of using these terms under the non-equilibrium hypoosmotic condition are now acknowledged.

**Comment:** Reduce repetition of Results in the Discussion.

**Response:** The Discussion was revised to emphasize interpretation and comparison with prior work rather than repeating numerical results.

**Comment:** Correct minor grammatical and formatting issues.

**Response:** The manuscript was edited for grammar, punctuation, terminology, units, hyphenation, and consistency.

# Reviewer 2

**Comment:** The Introduction could be restructured and some parts reconsidered for consistency.

**Response:** The Introduction was reorganized to provide a clearer progression from the practical importance of bovine semen cryopreservation, through the multifactorial determinants of post-thaw sperm quality, to the specific role of osmotic water transport and the limitations of previous stopped-flow studies. Several sentences were also revised for consistency and readability.

**Comment:** Page 2, lines 29–32: Recast the statements. Avoid writing “we found.”

**Response:** The relevant Abstract statements were recast using objective scientific phrasing, including “no significant differences were detected,” “direct association analyses identified,” and “significant volume rectification was observed.”

**Comment:** Add a table of motility and motion characteristics and sperm attributes of frozen bull spermatozoa.

**Response:** We thank the reviewer for this suggestion. The present study was specifically designed to evaluate post-thaw total and progressive motility in relation to osmotic-response parameters and hydraulic conductivity. Detailed CASA motion characteristics and additional sperm attributes were not predefined study outcomes and were not retained in the analytical dataset. We therefore did not add a table containing additional traits, because doing so would extend the manuscript beyond its predefined scope and could imply that these variables were systematically analyzed. The Methods now state clearly that total and progressive motility were the post-thaw motility outcomes evaluated.

**Comment:** Older references, including Lunstra and Echternkamp 1982, Gilmore et al. 1998, Noiles et al. 1993, Devireddy et al. 1999, and Liu and Foote 1998, may be replaced.

**Response:** We carefully reassessed these references. They were retained where they report original experimental observations and foundational quantitative data on sperm membrane permeability, cryoprotectant transport, osmotic tolerance, or bull reproductive physiology. In several instances, the specific numerical values and mechanistic observations discussed in the manuscript originate directly from these primary studies and cannot be accurately attributed to newer publications. To provide an updated context, we incorporated recent studies already cited in the manuscript and additional relevant references recommended during peer review. We believe that retaining foundational primary studies while adding current literature provides the most accurate and balanced representation of the field.

# Summary of Major Revisions

- Revised the manuscript title to emphasize implications for cryopreservation rather than presupposing a correlation.
- Restructured the Introduction and added relevant literature on oxidative stress and membrane integrity.
- Clarified the handling of individual and pooled ejaculates and the potential averaging effect of pooling.
- Strengthened the rationale for the 20 s hypoosmotic stopped-flow recording period.
- Added direct Pearson and Spearman correlations, standardized regression analyses, false discovery rate adjustment, and sensitivity power analysis.
- Expanded PCA reporting with explained variance and numerical loadings.
- Added Supplementary Tables S4–S8 and updated the Supporting Information description.
- Moderated claims regarding prediction and cryopreservation optimization.
- Clarified the indirect mechanistic value of Lp for membrane transport and osmotic tolerance.
- Standardized terminology and corrected grammatical, typographical, and formatting issues.

We again thank the Academic Editor and reviewers for their constructive comments, which substantially improved the manuscript.
