# Supplementary material for: Hyper- and hypoosmotic water permeability of bovine sperm as a function of age, breed, and ejaculate number: Implications for cryopreservation
Source: PLoS One. 2026 Sep 28;21(9):e0358553. doi: 10.1371/journal.pone.0358553 (PMC13618955; doi:10.1371/journal.pone.0358553)
Supplement: Peer review history

# Peer review: Decision Letter 0

**DOI:** 10.1371/journal.pone.0358553.r001

**Authors:** Muhammad Usman Mehmood

28 Jun 2026

Dear Dr. Benson,

Thank you for submitting your manuscript to PLOS ONE. After careful consideration, we feel that it has merit but does not fully meet PLOS ONE’s publication criteria as it currently stands. Therefore, we invite you to submit a revised version of the manuscript that addresses the points raised during the review process.

**ACADEMIC EDITOR:**

In light of the feedback provided by both reviewers, I kindly ask for a response to their comments. It seems essential to include a clear write-up and sample size, power analysis to complete with justification.

Please submit your revised manuscript by Aug 27 2026 11:59PM. If you will need more time than this to complete your revisions, please reply to this message or contact the journal office at plosone@plos.org. When you're ready to submit your revision, log on to https://www.editorialmanager.com/pone/ and select the 'Submissions Needing Revision' folder to locate your manuscript file.

- A letter that responds to each point raised by the academic editor and reviewer(s). You should upload this letter as a separate file labeled 'Response to Reviewers'.
- A marked-up copy of your manuscript that highlights changes made to the original version. You should upload this as a separate file labeled 'Revised Manuscript with Track Changes'.
- An unmarked version of your revised paper without tracked changes. You should upload this as a separate file labeled 'Manuscript'.

If you would like to make changes to your financial disclosure, please include your updated statement in your cover letter. Guidelines for resubmitting your figure files are available below the reviewer comments at the end of this letter.

If applicable, we recommend that you deposit your laboratory protocols in protocols.io to enhance the reproducibility of your results. Protocols.io assigns your protocol its own identifier (DOI) so that it can be cited independently in the future. For instructions see: https://journals.plos.org/plosone/s/submission-guidelines#loc-laboratory-protocols. Additionally, PLOS ONE offers an option for publishing peer-reviewed Lab Protocol articles, which describe protocols hosted on protocols.io. Read more information on sharing protocols at https://plos.org/protocols?utm_medium=editorial-email&utm_source=authorletters&utm_campaign=protocols.

As the corresponding author, your ORCID iD is verified in the submission system and will appear in the published article. PLOS supports the use of ORCID, and we encourage all coauthors to register for an ORCID iD and use it as well. Please encourage your coauthors to verify their ORCID iD within the submission system before final acceptance, as unverified ORCID iDs will not appear in the published article. *Only*  the individual author can complete the verification step; PLOS staff *cannot*  verify ORCID iDs on behalf of authors.

We look forward to receiving your revised manuscript.

Kind regards,

Muhammad Usman Mehmood

Academic Editor

PLOS One

Journal Requirements:

When submitting your revision, we need you to address these additional requirements.

1. Please ensure that your manuscript meets PLOS ONE's style requirements, including those for file naming. The PLOS ONE style templates can be found at

https://journals.plos.org/plosone/s/file?id=wjVg/PLOSOne_formatting_sample_main_body.pdf and

https://journals.plos.org/plosone/s/file?id=ba62/PLOSOne_formatting_sample_title_authors_affiliations.pdf.

2. Thank you for stating in your Funding Statement:

“The authors also acknowledge funding support from the National Science and Engineering Research Council (RGPIN-2023-04007 and ALLRP 585227 – 23 to JB)”

Please provide an amended statement that declares *all* the funding or sources of support (whether external or internal to your organization) received during this study, as detailed online in our guide for authors at http://journals.plos.org/plosone/s/submit-now.  Please also include the statement “There was no additional external funding received for this study.” in your updated Funding Statement.

Please include your amended Funding Statement within your cover letter. We will change the online submission form on your behalf.

3. Thank you for stating the following financial disclosure:

“The authors also acknowledge funding support from the National Science and Engineering Research Council (RGPIN-2023-04007 and ALLRP 585227 – 23 to JB)”’

Please state what role the funders took in the study.  If the funders had no role, please state: "The funders had no role in study design, data collection and analysis, decision to publish, or preparation of the manuscript."

If this statement is not correct you must amend it as needed.

Please include this amended Role of Funder statement in your cover letter; we will change the online submission form on your behalf.

4. Thank you for stating the following in the Acknowledgments Section of your manuscript:

“The authors also acknowledge funding support from the National Science and Engineering Research Council (RGPIN-2023-04007 and ALLRP 585227 – 23 to JB).”

We note that you have provided additional information within the Acknowledgements Section that is not currently declared in your Funding Statement. Please note that funding information should not appear in the Acknowledgments section or other areas of your manuscript. We will only publish funding information present in the Funding Statement section of the online submission form.

Please remove any funding-related text from the manuscript and let us know how you would like to update your Funding Statement. Currently, your Funding Statement reads as follows:

“The authors also acknowledge funding support from the National Science and Engineering Research Council (RGPIN-2023-04007 and ALLRP 585227 – 23 to JB).”

Please include your amended statements within your cover letter; we will change the online submission form on your behalf.

5. Your ethics statement should only appear in the Methods section of your manuscript. If your ethics statement is written in any section besides the Methods, please move it to the Methods section and delete it from any other section. Please ensure that your ethics statement is included in your manuscript, as the ethics statement entered into the online submission form will not be published alongside your manuscript.

6. If the reviewer comments include a recommendation to cite specific previously published works, please review and evaluate these publications to determine whether they are relevant and should be cited. There is no requirement to cite these works unless the editor has indicated otherwise.

Additional Editor Comments (if provided):

In light of the feedback provided by both reviewers, I kindly ask for a response to their comments. It seems essential to include a clear write-up and sample size, power analysis to complete with justification.

[Note: HTML markup is below. Please do not edit.]

Reviewers' comments:

Reviewer's Responses to Questions

**Comments to the Author**

1. Is the manuscript technically sound, and do the data support the conclusions?

Reviewer #1: Yes

Reviewer #2: Yes

**********

2. Has the statistical analysis been performed appropriately and rigorously?

Reviewer #1: Yes

Reviewer #2: Yes

**********

3. Have the authors made all data underlying the findings in their manuscript fully available?

The PLOS Data policy

Reviewer #1: Yes

Reviewer #2: Yes

**********

4. Is the manuscript presented in an intelligible fashion and written in standard English?

Reviewer #1: Yes

Reviewer #2: Yes

**********

Reviewer #1: This is a technically rigorous and well-executed study that employs stopped-flow fluorometry to quantify hydraulic conductivity (Lp) of bovine sperm under hypo- and hyperosmotic conditions across ejaculate number, breed, and age groups. The manuscript provides valuable data on volume rectification (higher Lp in hypertonic conditions) and demonstrates the utility of log-logistic modeling for permeability parameters, though the primary hypothesis of a correlation between fresh semen water permeability and post-thaw motility was not supported. The work is clearly written with strong methodological detail and appropriate statistical analyses, making it suitable for PLOS ONE after addressing gaps in literature integration, clarification of negative results, and minor grammatical refinements.

Major Concerns

Abstract

• The abstract effectively summarizes key findings but overstates the predictive utility of log-logistic and linear models for cryopreservation refinements without sufficient supporting data on improved outcomes.

• Clarify that the lack of correlation applies specifically to post-thaw motility under the study's freezing protocol, as this is a central negative result that should be highlighted upfront.

Introduction

• The background on cryopreservation challenges and osmotic stress is appropriate, but the transition to water permeability's role could better acknowledge membrane integrity factors during freezing. In the sentence discussing variability in post-thaw motility and the hypothesized role of water permeability, reference "Caffeic acid improves microscopic sperm parameters and antioxidant status of buffalo (Bubalus bubalis) bull semen following freeze-thawing process (DOI: 10.1016/j.cryobiol.2020.06.010)" should be included, as it provides supporting evidence on oxidative stress and membrane-related parameters affecting post-thaw sperm quality in bulls.

• The paragraph on previous stopped-flow studies (Chaveiro et al.) is good, but expand on why larger, multi-factorial studies are needed. In the sentence noting limitations of prior work with limited Holstein bulls, reference "The influence of L-proline and fulvic acid on oxidative stress and semen quality of buffalo bull semen following cryopreservation" should be included, as it demonstrates comparable variability in cryopreservation outcomes linked to membrane protectants and oxidative status.

Methods

• Bull selection and semen processing are well-described, but justification for pooling ejaculates after permeability testing (while analyzing separately) requires clearer rationale for potential bias. In the sentence detailing separate analysis of Ejaculate 1 and 2 before pooling, reference "The effects of glutathione supplementation on post-thawed Turkey semen quality and oxidative stress parameters and fertilization, and hatching potential (DOI: 10.1016/j.theriogenology.2021.11.010)" should be included, as it supports methods involving ejaculate variability and antioxidant-related membrane stability in cryopreservation.

• The stopped-flow protocol and volume calculations are detailed, but the choice of 20s recording for hypoosmotic conditions (not reaching equilibrium) needs stronger justification or sensitivity analysis. In the sentence explaining interpretation of 150 mOsm data as dynamic response, reference "Effects of bisphenol-S low concentrations on oxidative stress status and in vitro fertilization potential in mature female mice (PMID: 29326794)" should be included, since it addresses osmotic and oxidative impacts on gamete membrane function in related reproductive models.

• Statistical section is comprehensive but could specify power analysis for detecting correlations, given the negative result.

Discussion

• Interpretation of volume rectification is insightful, but the lack of Lp-motility correlation requires deeper exploration of alternative factors (e.g., oxidative stress during freezing). In the sentence discussing implications for cryopreservation optimization despite no direct correlation, reference "The effects of varying concentrations of glutathione and trehalose in improving microscopic and oxidative stress parameters in Turkey semen during liquid storage at 5 °C" should be included, as it offers complementary evidence on antioxidant modulation of membrane integrity and post-thaw quality.

• Age and breed effects on motility are well-noted, but mechanistic links to Lp could be strengthened. In the paragraph on ejaculate number and post-thaw differences, reference "Carob (Ceratonia siliqua L.) fruit hydro-alcoholic extract alleviates reproductive toxicity of lead in male mice: Evidence on sperm parameters, sex hormones, oxidative stress biomarkers and expression of Nrf2 and iNOS (PMCID: PMC6941692)" should be included, as it provides supporting data on factors influencing sperm membrane resilience under stress.

• Clinical/translational implications for AI centers are present but could more explicitly address why Lp may still be useful indirectly (e.g., via membrane fluidity).

Results

• Figures are generally clear, but some boxplots (e.g., Fig. 7) would benefit from explicit annotation of percentage differences in Lp for quick reader comprehension.

• PCA and regression results are strong but should better address why motility parameters cluster near origin (low contribution).

Minor Concerns

Abstract

• Minor grammatical issue: "its correlation with post-thaw motility" in title/short title could be "and its correlation..." for parallelism.

• Ensure consistent use of "hypoosmotic" vs. "hypotonic" throughout.

Introduction

• Some sentences are lengthy; split the one on Gilmore et al. and Agca et al. for better readability.

• Minor: "Chaviero et al." should consistently be "Chaveiro et al." (check spelling).

Methods

• In CFDA loading: "alloquats" is a typo for "aliquots".

• Several long compound sentences in stopped-flow and modeling sections could be broken for clarity.

• "π0" and "πf" notation is clear but ensure all equations are properly rendered in final PDF.

Discussion

• Minor repetition of motility findings from Results; condense.

• Ensure consistent subscript formatting for Lp and osmolality terms.

• The final sentence on modeling insights is strong but could tie back more directly to the negative correlation finding.

Reviewer #2: Title: Hyper- and hypoosmotic water permeability of bovine sperm as a function of age, breed, and ejaculate number and its correlation with post-thaw motility

Article Type: Research Article

Manuscript Id: PONE-D-26-30013

Comments:

The authors have provided information pertaining to Hyper- and hypoosmotic water permeability of bovine sperm as a function of age, breed, and ejaculate number and its correlation with post-thaw motility. The introduction could be restructured and some parts are reconsidered for consistency.

Page 2, lines 29-32, Recast the statements. Avoid writing we found-----

Add a table of motility and motion characteristics and sperm attributes of frozen bull spermatozoa.

Too old references viz. Lunstra and Echternkamp 1982; Gilmore et al 1998; Noiles et al 1993; Devireddy et al 1999; Gilmore et al 1998; Liu and Foote 1998 may be replaced.

**********

what does this mean?). If published, this will include your full peer review and any attached files.

If you choose “no”, your identity will remain anonymous but your review may still be made public.

**Do you want your identity to be public for this peer review?**  For information about this choice, including consent withdrawal, please see our Privacy Policy

Reviewer #1: No

Reviewer #2: No

**********

[NOTE: If reviewer comments were submitted as an attachment file, they will be attached to this email and accessible via the submission site. Please log into your account, locate the manuscript record, and check for the action link "View Attachments". If this link does not appear, there are no attachment files.]

To ensure your figures meet our technical requirements, please review our figure guidelines: https://journals.plos.org/plosone/s/figures

You may also use PLOS’s free figure tool, NAAS, to help you prepare publication quality figures: https://journals.plos.org/plosone/s/figures#loc-tools-for-figure-preparation.

NAAS will assess whether your figures meet our technical requirements by comparing each figure against our figure specifications.

# Author comment: Author response to Decision Letter 1

**DOI:** 10.1371/journal.pone.0358553.r002

4 Aug 2026

We thank the editor and reviewers for their constructive comments. We have made efforts to carefully address each comment and have outlined these changes and our responses in the attached "Response to Reviewers" document. We look forward to continued feedback on our article.

Attachment Submitted filename: Response to Reviewers.docx

# Editor report: Decision Letter 1

**DOI:** 10.1371/journal.pone.0358553.r003

**Authors:** Muhammad Usman Mehmood

3 Sep 2026

Hyper- and hypoosmotic water permeability of bovine sperm as a function of age, breed, and ejaculate number: implications for cryopreservation.

PONE-D-26-30013R1

Dear Dr. Mohsen Sharafi,

We’re pleased to inform you that your manuscript has been judged scientifically suitable for publication and will be formally accepted for publication once it meets all outstanding technical requirements.

Within one week, you’ll receive an e-mail detailing the required amendments. When these have been addressed, you’ll receive a formal acceptance letter and your manuscript will be scheduled for publication.

An invoice will be generated when your article is formally accepted. Please note, if your institution has a publishing partnership with PLOS and your article meets the relevant criteria, all or part of your publication costs will be covered. Please make sure your user information is up-to-date by logging into Editorial Manager at Editorial Manager® and clicking the ‘Update My Information' link at the top of the page. For questions related to billing, please contact billing support.

If your institution or institutions have a press office, please notify them about your upcoming paper to help maximize its impact. If they’ll be preparing press materials, please inform our press team as soon as possible -- no later than 48 hours after receiving the formal acceptance. Your manuscript will remain under strict press embargo until 2 pm Eastern Time on the date of publication. For more information, please contact onepress@plos.org.

Kind regards,

Muhammad Usman Mehmood

Academic Editor

PLOS One

Additional Editor Comments (optional):

Thank you very much. The fundamental revisions have been completed, and I sincerely appreciate the authors’ thorough revisions and efforts to improve the manuscript.

Reviewers' comments:

# Editor report: Acceptance letter

**DOI:** 10.1371/journal.pone.0358553.r004

**Authors:** Muhammad Usman Mehmood

PONE-D-26-30013R1

PLOS One

Dear Dr. Benson,

I'm pleased to inform you that your manuscript has been deemed suitable for publication in PLOS One. Congratulations! Your manuscript is now being handed over to our production team.

At this stage, our production department will prepare your paper for publication. This includes ensuring the following:

* All references, tables, and figures are properly cited

* All relevant supporting information is included in the manuscript submission,

* There are no issues that prevent the paper from being properly typeset

You will receive further instructions from the production team, including instructions on how to review your proof when it is ready. Please keep in mind that we are working through a large volume of accepted articles, so please give us a few days to review your paper and let you know the next and final steps.

Lastly, if your institution or institutions have a press office, please let them know about your upcoming paper now to help maximize its impact. If they'll be preparing press materials, please inform our press team within the next 48 hours. Your manuscript will remain under strict press embargo until 2 pm Eastern Time on the date of publication. For more information, please contact onepress@plos.org.

You will receive an invoice from PLOS for your publication fee after your manuscript has reached the completed accept phase. If you receive an email requesting payment before acceptance or for any other service, this may be a phishing scheme. Learn how to identify phishing emails and protect your accounts at https://explore.plos.org/phishing.

If we can help with anything else, please email us at customercare@plos.org.

Thank you for submitting your work to PLOS One and supporting open access.

Kind regards,

PLOS One Editorial Office Staff

on behalf of

Dr. Muhammad Usman Mehmood

Academic Editor

PLOS One
